# Effects of auditory stimuli during exhaustive exercise on cerebral oxygenation and psychophysical responses

**DOI:** 10.1162/IMAG.a.1166

**Published:** 2026-03-20

**Authors:** Ségolène M.R. Guérin, Costas I. Karageorghis, Marine R. Coeugnet, Marcelo Bigliassi, Yvonne N. Delevoye-Turrell

**Affiliations:** Univ. Littoral Côte d’Opale, Univ. Artois, Univ. Lille, ULR 7369 – URePSSS – Unité de Recherche Pluridisciplinaire Sport Santé Société, Calais, France; Brunel University of London, Uxbridge, Middlesex, United Kingdom; Univ. Lille, UMR 9193 – SCALab – Sciences Cognitives et Sciences Affectives, Lille, France; Florida International University, Miami, FL, United States

**Keywords:** cerebral oximetry, cycling, physical activity, prefrontal activity, ventilatory threshold

## Abstract

Asynchronous music has been commonly used to reduce perceived exertion and render the exercise experience more pleasant. Research has indicated that in-task asynchronous music can reallocate an individual’s attentional focus to task-unrelated signals and increase the use of dissociative thoughts. Nonetheless, the brain mechanisms that underlie the purported benefits of music during exercise remain largely unknown due to the severe motion-related restrictions of popular neuroimaging techniques. Functional near-infrared spectroscopy (*f*NIRS) represents a noninvasive imaging method that is particularly suited to exercise-related protocols given its high tolerance to motion artifacts. With use of *f*NIRS, the purpose of the present study was to determine the point of onset of cerebral oxygenation decline during exercise, and how this is influenced by the presence of asynchronous (ambient) motivational music. A continuous-wave *f*NIRS system was used to record the prefrontal, motor, and parietal hemodynamic responses of 36 participants (*M*_age_ = 23.1 years; 17 females, 16 males) who performed a cycle ergometry exercise protocol to the point of volitional exhaustion. Results indicated that asynchronous music did not engender any significant changes in cerebral hemodynamics, exercise endurance, or subjective measures, when compared with audiobook and silence control conditions. A nonsignificant trend emerged, suggesting reduced medial prefrontal cortex activation and slightly improved endurance with music. The present findings highlight the complexities associated with the influence of music on exercise-related brain activity. Further research employing more homogeneous samples and alternative exercise protocols is warranted to elucidate the neurophysiological mechanisms that underlie the effects of music during exhaustive exercise.

## 1. Introduction

It is widely recognized that music and physical activity have a close connection (for a review, see Kuan et al., 2026). This relationship has been fueled by rapid development in the digital technology that underlies music delivery and a growing recognition that well-selected music can enhance the experience of physical activity (Terry et al., 2020). In the exercise domain, music is used to partially block negative bodily signals from entering focal awareness, elevate affective states, and provide a rhythmic cue that can prolong physical effort (Bigliassi et al., 2017; Karageorghis et al., 2018).

In the exercise context, an ergogenic aid can be broadly defined as a technique or substance used for the purpose of enhancing or prolonging performance (Thein et al., 1995). Music is an oft-used ergogenic aid in this context (see Karageorghis, 2020, for a review). During an exercise task, there are two main ways in which music can be applied: synchronously and asynchronously. The phenomenon observed when exercisers synchronize their movements with the rhythmical qualities of music is commonly referred to as auditory–motor synchronization (Karageorghis & Terry, 1997). In recent years, two main forms of auditory–motor synchronization have been proposed: (a) *active synchronization*, in which individuals consciously synchronize their movement rate with the music tempo and (b) *passive synchronization*, in which the music tempo is automatically adjusted to match the movement rate of the exerciser (Karageorghis, 2020). The application of asynchronous or ambient music, by way of contrast, does not involve synchronization between an exerciser’s movements and the rhythmical qualities of a piece of music. Asynchronous music represents the most widely used form of music application during individual exercise routines (Karageorghis, 2020).

Asynchronous music has been commonly used to reduce perceived exertion and render the exercise experience more pleasant (Karageorghis et al., 2017; Kawabata & Chua, 2021). Collectively, studies have indicated that in-task asynchronous music can reallocate an individual’s attentional focus to task-unrelated signals, increase the frequency of dissociative thoughts, and consequently ameliorate the effects of fatigue-related symptoms (e.g., limb discomfort, increased respiration rate; Bigliassi et al., 2018; Karageorghis & Priest, 2012). Jones et al. (2014) reported that even high-intensity exercise performed at 5% above the first ventilatory threshold (i.e., the point during exercise at which breathing becomes labored) is rendered more pleasant by the presence of asynchronous music. In the present study, music was applied in the asynchronous mode during exercise at a constant workload at 5% above the first ventilatory threshold (VT1) that concludes with volitional exhaustion.

A clutch of studies has indicated that music-induced cerebral phenomena may contribute to exercise performance (for a review, see Karageorghis, 2020). Through adjustments of neural dynamics, music-related interventions were found to guide attention away from the unpleasant sensations caused by exercise-related tasks (Bigliassi et al., 2016, 2019). Reallocating attention outwardly during exercise was associated with reduced frontal–central connectivity (Bigliassi et al., 2017) and increased activation of the left inferior frontal gyrus (Bigliassi et al., 2018). Furthermore, the parietal cortex was found to be implicated in the conscious awareness of bodily sensations through neural inputs from thalamocortical neurones (Crossman & Neary, 2014). Most of the aforementioned electroencephalogram (EEG) and functional magnetic resonance imaging (*f*MRI) studies used relatively simple motor tasks (e.g., isometric handgrip, ankle-dorsiflexion task) that are somewhat disconnected from ecological physical activities (e.g., cycling, running). This is due mainly to the severe motion-related methodological restrictions of current brain-imaging technologies (Karageorghis et al., 2018).

A neuroimaging technique used to assess brain metabolism is functional near-infrared spectroscopy (*f*NIRS), which entails a noninvasive imaging method that quantifies chromophore concentration resolved from the measurement of near-infrared light attenuation, temporal or phasic changes (Stute et al., 2025). This technique is particularly salient to exercise-related protocols given its high tolerance for motion artifacts (Leff et al., 2011; Pinti et al., 2023). In addition, the neurophysiological mechanisms that underlie the influence of attentional manipulation on tissue oxygenation during exercise can be investigated with an acceptable degree of temporal resolution (Vitorio et al., 2017).

*f*NIRS is a technique that has proven to be effective in the examination of cortical oxygenation during exercise (Herold et al., 2017; Jones & Wheat, 2023; Wang et al., 2025). Notably, performance of a long-duration, constant-load cycling task increased prefrontal (i.e., medial prefrontal cortex [mPFC] and dorsolateral prefrontal cortex [dlPFC]) oxygenation that became stable over time (Tempest et al., 2017). Similar results were reported by Jones and Ekkekakis (2019) across the dlPFC during incremental (14-W/min ramp) recumbent cycling. Specifically, these authors showed that higher levels of right dlPFC oxygenation were associated with lower ratings of affective valence for participants who reported a preference for low-intensity exercise. They suggested that the observed dlPFC activity was associated with the cognitive regulation of unpleasant affective responses to incremental exercise. This was experienced to a greater degree by participants with low preference-for-exercise levels when compared with their high-preference-for-exercise counterparts.

The sensation of discomfort and pain is often an indication to the organism that exercise should be surceased. These signals become more intense at the respiratory compensation point or ventilatory threshold 2 (VT2); the moment during exercise at which minute ventilation starts to become excessive in relation to exhaled carbon dioxide. Studies that have used *f*NIRS to evaluate mPFC and dlPFC hemodynamics have reported a decrease in cerebral oxygenation at intensities above VT2 (e.g., Ochi et al., 2018; Rupp & Perrey, 2008). The reduced availability of oxygen in the brain might influence central nervous system motor output, and constitutes a signal that eventually leads to a sharp degradation in exercise performance.

The cerebral hemodynamic phenomena that are observed during high-intensity exercise have blood-related concomitants, insofar as blood pH is reduced with the onset of anaerobic metabolism (Bhambhani et al., 2007). Specifically, after VT2, there is a decrease in the partial pressure of arterial carbon dioxide (${\text{PaCO}}_{2}$), given that exercise-induced hypocapnia results in cerebral vasoconstriction. The upshot of this is reduced cerebral oxygenation. Nonetheless, the directionality of the relationship between cerebral hemodynamics and ${\text{PaCO}}_{2}$ is presently only a matter for speculation (i.e., whether it is a cerebral mechanism that instigates the onset of lactic acid generation; c.f. Quistorff et al., 2008). The patterns of cerebral (de)oxygenation observed in the PFC at the VT2 are associated with the neural control of the musculature as well as processing of emotions, thoughts, and afferent feedback from the working muscles and internal organs (Robertson & Marino, 2016). In order to counteract the effects of fatigue, the dlPFC relies on motivational signals traveling through the mesocortical and mesolimbic systems. Insufficient motivation (either conscious or unconscious) to maintain movement execution is likely to cause deoxygenation of the central executive network and task disruption (Bigliassi et al., 2022). At acute levels of brain deoxygenation, the organism is driven toward the discontinuation of exercise (Ekkekakis, 2009; Perrey, 2008).

**Fig. 1.**
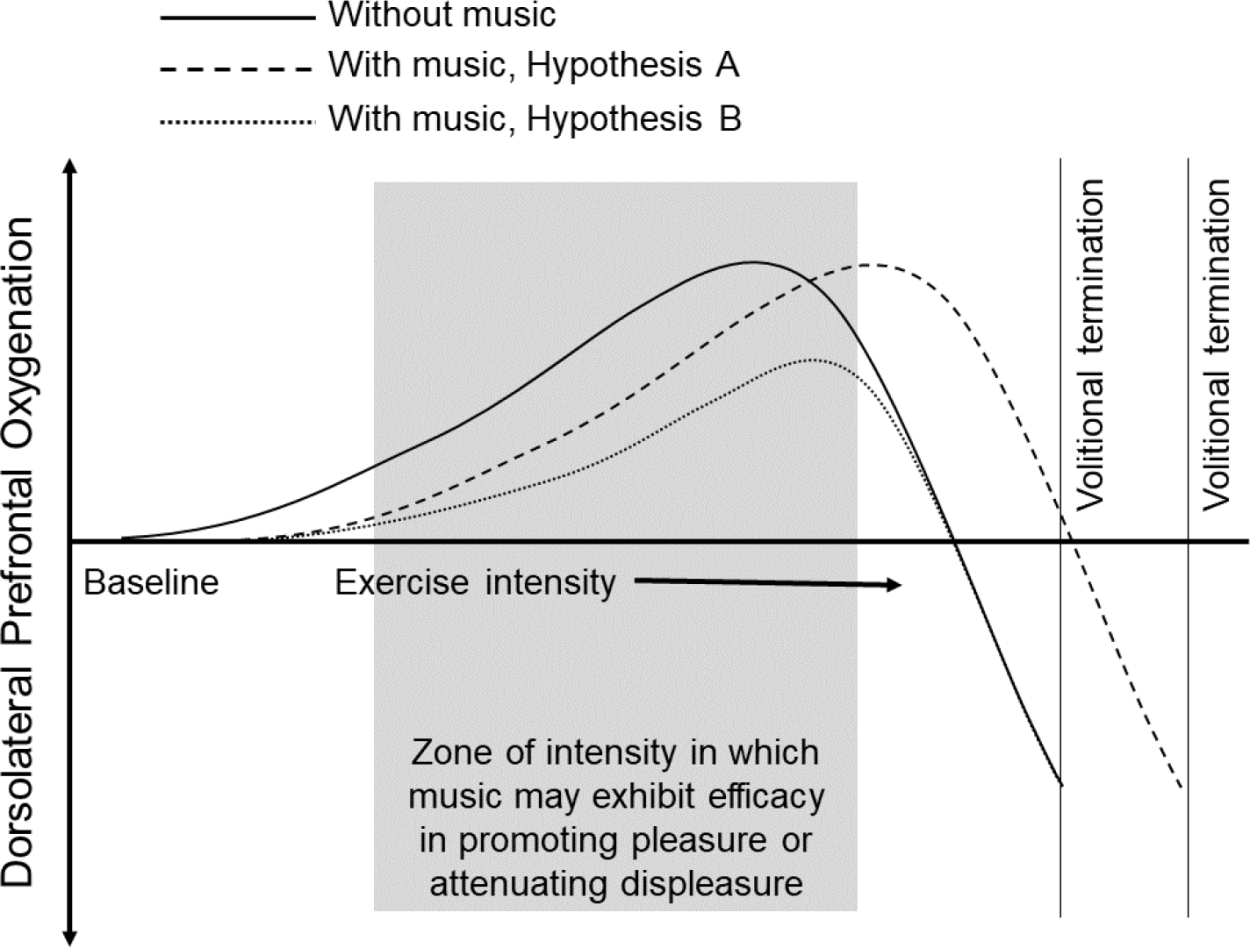
Schematic representation of the hypothetical neurophysiological mechanisms underlying the effect of music during exercise. *Note.* Reproduced from Karageorghis, C. I., Ekkekakis, P., Bird, J. M., & Bigliassi, M. (2017). Music in the exercise and sport domain: Conceptual approaches and underlying mechanisms. In M. Lesaffre, P.-J. Maes, & M. Leman (Eds.), *The Routledge companion to embodied music interaction* (p. 288). https://doi.org/10.4324/9781315621364. Copyright 2017 by Routledge. Reprinted with permission through PLSclear.

Music can be used to prolong physical effort, possibly through the neurophysiological effects that it has at, or close to, the RCP (Bigliassi et al., 2017; Karageorghis et al., 2018). Two hypotheses have been offered to account for the neurophysiological mechanisms that underlie the effects of music during exercise and physical activity: (a) music delays the decrease in prefrontal oxygenation and shifts “the entire oxygenation curve toward higher levels of exercise intensity” (Karageorghis, 2020, p. 942); (b) music delays the increase in prefrontal oxygenation due to a reallocation of attention toward exteroceptive cues (Karageorghis et al., 2017; see Fig. 1). Notably, Jones and Ekkekakis (2019) reported an increase in dlPFC oxygenation over time during recumbent cycling, but no such difference was observed between a music condition and a no-music control. In this study, however, participants did not continue cycling until volitional exhaustion, but stopped after 15 min. Accordingly, it is plausible that, rather than attenuate prefrontal oxygenation, the application of music delayed the decline that accompanies volitional exhaustion.

## 2 Objectives and Hypotheses

The purpose of the present study was to determine the point of onset of cerebral oxygenation decline during an exercise protocol (at a constant workload of 5% above VT1) and how this is modulated by the presence of asynchronous music. More specifically, we assessed the effects of pleasurable auditory stimuli (i.e., music) on the cerebral oxygenation time course during a constant-rate cycle ergometry exercise task to exhaustion, commencing at 5% above VT1. The task was executed under three conditions: asynchronous music, an audiobook control, and a no-audio control. The audiobook condition was included to control for the effects of auditory attentional distraction that is devoid of musical components (e.g., melody and harmony). Brain oxygenation was recorded using a continuous-wave *f*NIRS system over the bilateral mPFC, dlPFC, primary motor cortex, and lateral parietal cortex.

We hypothesized that the decrease in prefrontal (i.e., mPFC and dlPFC) oxygenation would be observed earlier under conditions in which participants exercise in silence or with an audiobook when compared with exposure to asynchronous motivational music ($H_{1}$). Exercise with music would lead to less prefrontal ($H_{2}$) and more parietal ($H_{3}$) activation when compared with exercising in silence or with an audiobook. In addition, as a sanity check for the effect of music exposure on prefrontal and parietal brain activity, we hypothesized that occipital cortex activation would not differ among the experimental conditions (i.e., negative control; $H_{4}$). We ran a series of pilot tests to confirm that the present experimental protocol was logistically feasible and that planned analyses allowed us to test the research hypotheses (see Methods section).

## 3 Methods

### 3.1 Participants

**Fig. 2.**
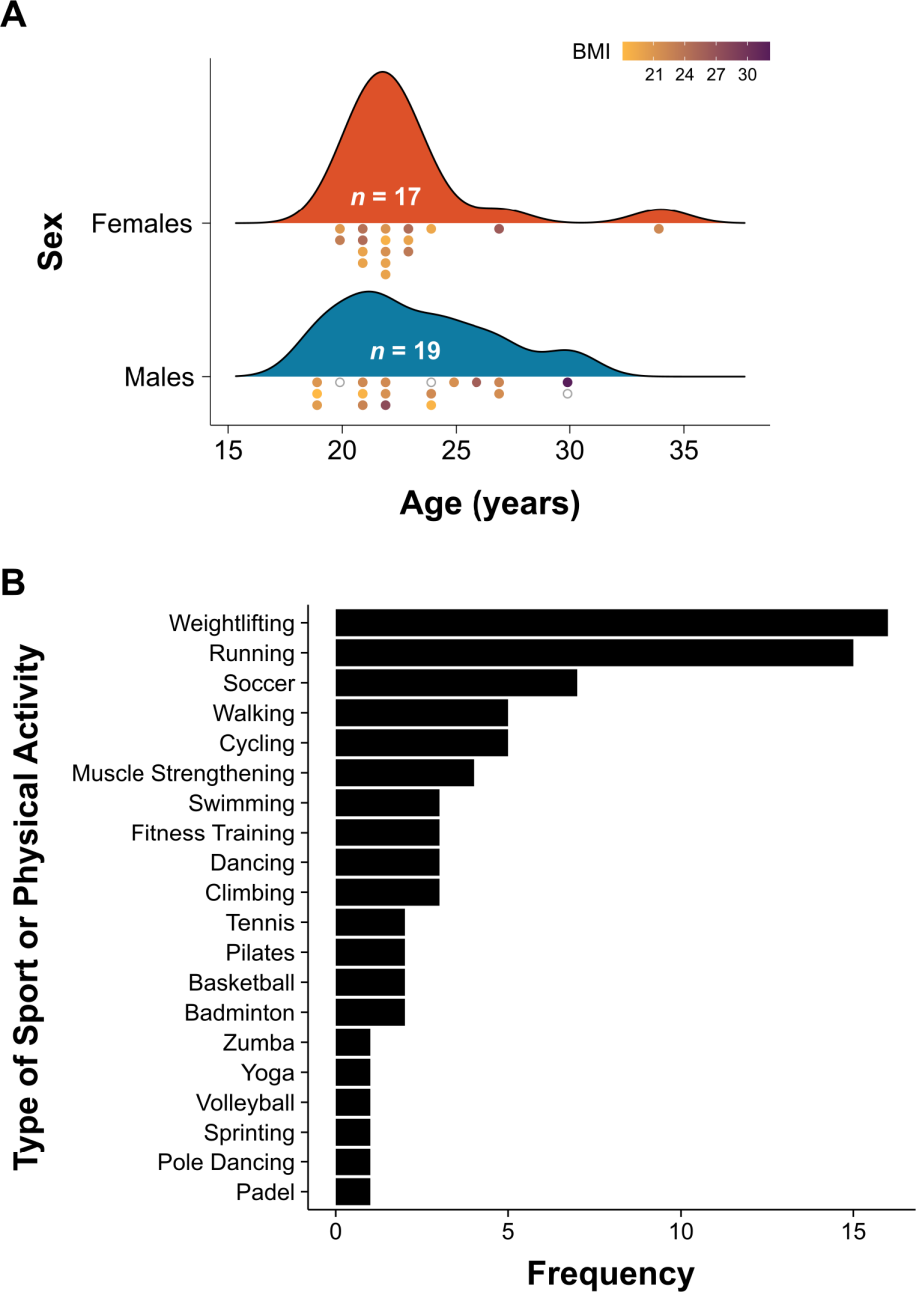
Participant demographic data. *Note.* (A) Age, sex, and BMI level of participants. (B) Background of participants in terms of sport and physical activity. BMI = body mass index. Each dot represents an individual participant. Empty gray circles represent participants for whom the BMI value is missing.

Volunteer adults were eligible if in the age range 18–35 years, recreationally active, and apparently healthy. Recreationally active is defined as those who engage in 45–90 min of moderate-intensity exercise (3–6 metabolic equivalents [METs]) 2–4 times a week over the previous 6 months (see Kelleher et al., 2010). To be included in the study, participants needed to have brought a recent (<12 months) medical certificate from their personal physician stating that they were fit to engage in high-intensity physical exercise. Participants were excluded from the study if they self-reported: (a) exercising >5 times per week at moderate intensity, (b) incidents of motor dysfunction, (c) hearing deficiency, (d) epilepsy, or (e) head trauma (i.e., loss of consciousness for more than 5 min). They were compensated for their time (i.e., €40 [US$48] for the completion of all four trials). Sample demographic details are shown in Figure 2.

The sample size for the critical statistical test of each research hypothesis was calculated using R with the “pwr” and “TOSTER” packages (the code is available here: https://doi.org/10.5281/zenodo.6261358). The required sample size has been computed for paired-samples *t* tests, which are the critical statistical tests (see Table 1). The *f*NIRS results of Ozawa et al. (2019) were used as a parameter for $H_{1}$–$H_{2}$ across the mPFC. For $H_{1}$–$H_{2}$ across the dlPFC and $H_{3}$, the *f*NIRS results of Oh et al. (2018) were used. For $H_{4}$, the *f*NIRS results of Guérin et al. (2021) were used. For $H_{1}$–$H_{2}$, the power analysis indicated that 30 participants would be required for the mPFC (*d* = 0.64; α = .02; 1-β = .90) and nine participants for the dlPFC (*d* = 1.38; α = .02; 1-β = .90). In addition, nine participants would be required for $H_{3}$ (*d* = 1.37; α = .02; 1-β = .90) and 36 participants for $H_{4}$ (*d* = 0.62; α = .02; 1-β = .90; see Table 1). Accordingly, a sample of 36 participants was recruited for the present study.

**Table 1.** Estimated required sample and effect sizes.

Question	Hypothesis	Sampling plan	Analysis plan	Rationale for deciding the sensitivity of the test for confirming or disconfirming the hypothesis	Interpretation given to different outcomes	Theory that could be shown wrong by the outcomes	Results
The decrease in prefrontal oxygenation will be observed earlier under conditions in which participants exercise in silence or with an audiobook, when compared with exposure to asynchronous motivational music.	$D_{\text{HbO2,mPFC}}$ will be larger during the music condition vs. the audiobook and silence conditions.	*N* = 30 (*d* = 0.64; α = .02; 1-β = .90)	Pairwise *t* tests	Small telescopes approach ($d_{\text{SESOI}}$ = 0.28)	The hypothesis will be accepted if the statistical test is significant (*p* < .020) and the associated Cohen’s *d* > $d_{\text{SESOI}}$ .	Karageorghis et al.’s (2017) Hypothesis A (see Fig. 1) logically extended to mPFC activity.	The hypothesis was not supported.
	$D_{\text{HbO2,dlPFC}}$ will be larger during the music condition vs. the audiobook and silence conditions.	*N* = 9 (*d* = 1.38; α = .02; 1-β = .90)	Pairwise *t* tests	Small telescopes approach ($d_{\text{SESOI}}$ = 0.38)		Karageorghis et al.’s (2017) Hypothesis A (see Fig. 1).	The hypothesis was not supported.
Less prefrontal activation will be observed when participants exercise with music, when compared with when they exercise in silence or with an audiobook.	$\beta_{\text{HbO2,mPFC}}$ will be larger during the audiobook and silence conditions vs. the music condition.	*N* = 30 (*d* = 0.64; α = .02; 1-β = .90)	Pairwise *t* tests	Small telescopes approach ($d_{\text{SESOI}}$ = 0.28)	The hypothesis will be accepted if the statistical test is significant (*p* < .020) and the associated Cohen’s *d* > $d_{\text{SESOI}}$ .	Role of the mPFC in appraisal and expression of negative emotions as proposed by Etkin et al. (2011).	The hypothesis was not supported.
	$\beta_{\text{HbO2,dlPFC}}$ will be larger during the audiobook and silence conditions vs. the music condition.	*N* = 9 (*d* = 1.38; α = .02; 1-β = .90)	Pairwise *t* tests	Small telescopes approach ($d_{\text{SESOI}}$ = 0.38)		Karageorghis et al.’s (2017) Hypothesis B (see Fig. 1).	The hypothesis was not supported.
Less parietal activation will be observed under conditions in which participants exercise in silence or with an audiobook, when compared with when they exercise with music.	$\beta_{\text{HbO2,lPC}}$ will be larger during the music condition vs. the audiobook and silence conditions.	*N* = 9 (*d* = 1.37; α = .02; 1-β = .90)	Pairwise *t* tests	Small telescopes approach ($d_{\text{SESOI}}$ = 0.38)	The hypothesis will be accepted if the statistical test is significant (*p* < .020) and the associated Cohen’s *d* > $d_{\text{SESOI}}$ .	Role of the parietal cortex to facilitate the selection of relevant signals proposed by Bigliassi (2021).	The hypothesis was not supported.
Similar hemodynamic responses of the occipital cortex will be observed across conditions.	$\beta_{\text{HbO2,motor}}$ will be similar during the music, audiobook, and silence conditions.	*N* = 36 (*d* = 0.62; α = .02; 1-β = .90)	TOSTs	Small telescopes approach ($d_{\text{SESOI}}$ = 0.62)	The hypothesis will be confirmed if both *t* tests are significant.	Not applicable (control condition).	The hypothesis was supported.

*Note.* Statistical power, planned analyses, and critical statistical tests for each research hypothesis. mPFC = medial prefrontal cortex; dlPFC = dorsolateral prefrontal cortex; lPC = lateral parietal cortex; RM ANOVA = repeated-measures analysis of variance; TOSTs = two one-sided *t* tests; SESOI = smallest effect size of interest.

The small telescopes approach was used to determine the smallest effect size of interest (SESOI; i.e., the difference that is considered too small to be meaningful; Simonsohn, 2015). Accordingly, the SESOI was set to the effect size that an earlier study would have had 33% power to detect (Lakens et al., 2018).^1^ In line with the studies used to derive the effect sizes for the power analysis, the *f*NIRS results of Ozawa et al. (2019) were used as parameters for $H_{1}$–$H_{\text{2}}$ across the mPFC, with a one-tailed test. For $H_{1}$–$H_{2}$ across the dlPFC and $H_{3}$, the *f*NIRS results of Oh et al. (2018) were used, with a one-tailed test. For $H_{4}$, the *f*NIRS results of Guérin et al. (2021) were used, with a two-tailed test. The SESOI computations were performed using R (the code is available as Supplementary Material here: https://doi.org/10.5281/zenodo.6261358) and the outputs are displayed in Table 1.

### 3.2 Experimental procedures

The study consisted of four sessions. There was a minimum recovery period of 48 h between sessions. Participants were advised to refrain from engaging in physical activity during the day of the experiment. They were also advised to avoid intense physical activity the day before the experiment.

Session 1 entailed screening, administration of questionnaires, and protocol habituation. Sessions 2–4 were administered in a fully counterbalanced order and comprise cycling (a) with asynchronous music (120–123 beats per minute [bpm]), (b) with an audiobook (audio control), (c) without any extraneous auditory stimuli (i.e., ambient noise control). The procedure used for the selection of motivational music tracks is presented in Supplementary File 1.

During Session 1, the participant read an information sheet and was afforded an opportunity to ask questions and sign an informed consent form. Participants performed an incremental ${\dot{\text{V}}\text{O}}_{\text{2max}}$ test on a cycle ergometer (Ergomedic 874E, Monark, Vansbro, Sweden) to determine a work rate representative of 5% above VT1 (for details on its determination, see Supplementary File 2). Five percent above VT1 was computed for each participant using the heart rate variability index of root mean square of successive differences (RMSSD; see Karapetian et al., 2008). Participants were also administered several questionnaires relating to (a) sociodemographic and anthropometric details, (b) self-reported physical activity level (International Physical Activity Questionnaire, IPAQ; Craig et al., 2003), (c) motivation to engage in physical activity (Behavioural Regulations in Exercise Questionnaire, BREQ-3; Markland & Tobin, 2004), and (d) tolerance of exercise intensity (Preference for and Tolerance of the Intensity of Exercise Questionnaire, PRETIE-Q; Carlier et al., 2017).

**Fig. 3.**
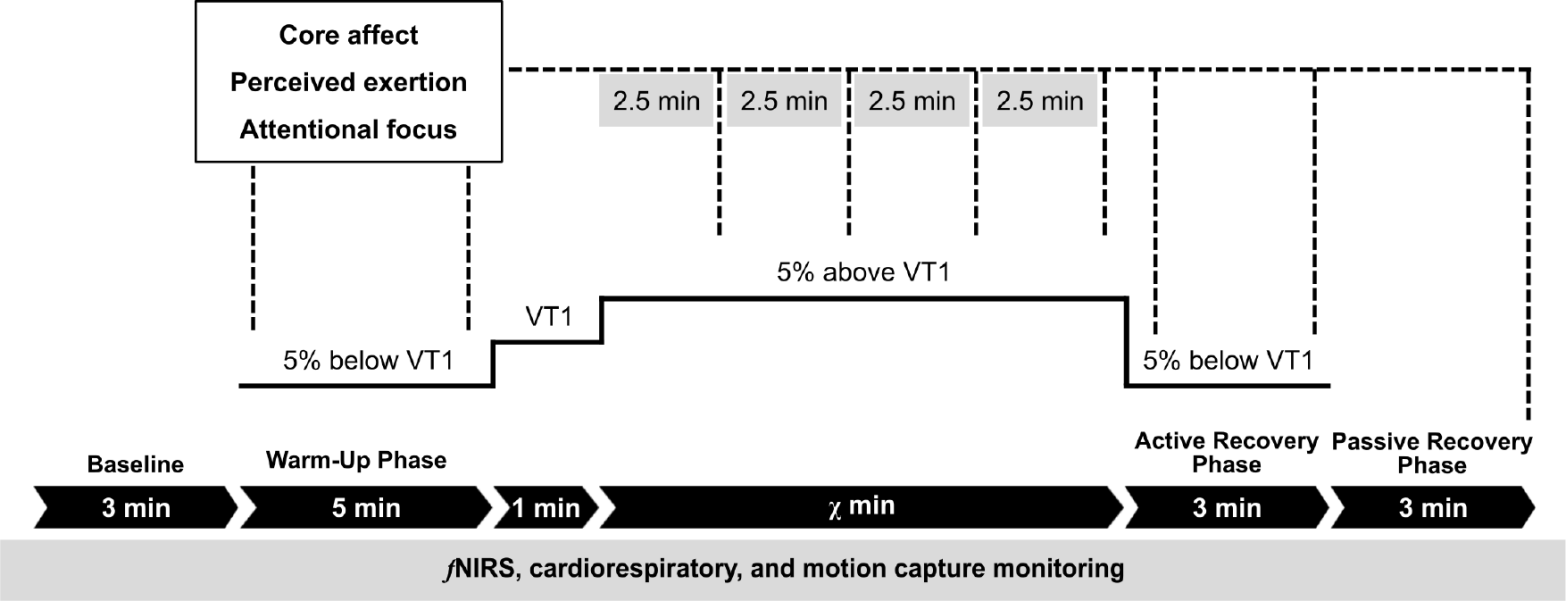
Experimental protocol of the present study. *Note.* VT1 = first ventilatory threshold.

During Sessions 2–4, participants underwent an exercise test on the cycle ergometer. The ambient temperature was controlled via use of a climate-control system to maintain $20^{\circ }\text{C}$ . Participants cycled at a constant rate of 63 rpm (revolutions per minute) to avoid synchronization of the pedal revolutions with the tempo of the music tracks (i.e., 120–123 bpm). After a 5-min warm up at 5% below VT1 and a 1-min transition phase performed at VT1, the resistance of the cycle ergometer was increased so that the participant exercised at 5% above VT1. For the experimental conditions, the auditory stimulus (i.e., asynchronous music or audiobook) was played to the participant from 1 min before the end of the warm-up session, up to the point at which they reach volitional exhaustion. The session was terminated when the participant was no longer able to maintain the prescribed pedal rate of 63 rpm for a period >10 s ^2^ (see Fig. 3). Thereafter, there was a 3-min active warm down at 63 rpm at an intensity of 5% below VT1.

### 3.3 Data acquisition and processing

#### 3.3.1 Questionnaires

Core affect (Feeling Scale and Felt Arousal Scale; Hardy & Rejeski, 1989; Svebak & Murgatroyd, 1985), perceived exertion (Borg Category Ratio-10 scale, CR10; Borg, 1982), and attentional focus (Attention Scale; Tammen, 1996) were assessed during the cycle ergometer exercise (i.e., at the beginning and end of warm up, every 2.5 min into the 5% above VT1 stage, at the beginning and end of the active recovery stage, and at the end of passive recovery; see Fig. 3). Physical activity enjoyment (Physical Activity Enjoyment Scale, PACES; Delignières & Perez, 1998) and remembered pleasure (visual analogue scale developed by Zenko et al., 2016) were assessed at the end of each experimental session.

#### 3.3.2 Cardiorespiratory monitoring

**Fig. 4.**
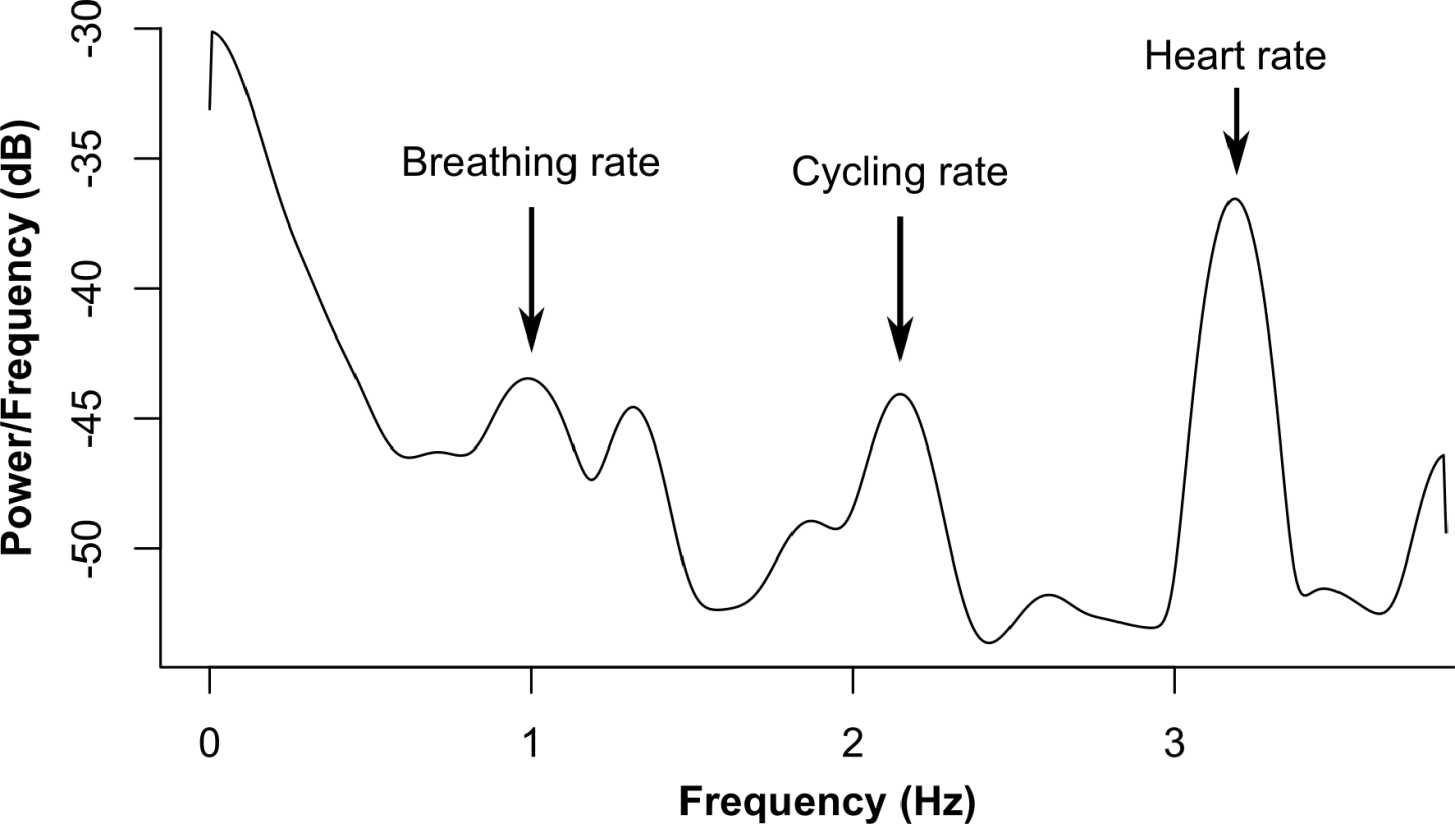
Welch power-spectral density of the raw *f*NIRS data. *Note.* These data were obtained from a pilot test.

Respiratory rate monitoring was facilitated by use of TSD201 respiratory effort transducer, connected to a MP150 Biopac device (Biopac Systems, Goleta, USA). This respiratory belt was placed around the chest wall, at the level of the sternum. During Sessions 2–4, heart rate was recorded by means of a BN-EL45-LEAD3 lead set and two disposable patch electrodes. The electrodes were placed on the participant’s right and left clavicles. Data acquisition was facilitated by the AcqKnowledge software that is included in the MP system. The sampling frequency was set to 250 Hz. Heart rate during Session 1 was assessed by means of a Polar system (H10 Polar strap) and the HRV Logger app (correction = workout). The *f*NIRS technique measures cerebral oximetry, which is strongly associated with respiratory and cardiac functioning (see Fig. 4; Pinti et al., 2019). Using spectral analysis (Welch’s estimation method), both heart and respiratory rates can be identified in the *f*NIRS signal. The ability to identify these two frequency components served to ensure the validity of *f*NIRS measures. More specifically, the presence of such physiological oscillations indicates that the acquired signals reflect genuine hemodynamic activity, rather than noise or artifacts.

#### 3.3.3 Head blood-volume pulse assessment

To control for extra-cerebral noise, noncortical hemodynamic responses were monitored by means of a photoplethysmograph sensor (Shimmer3 GSR+ unit; Shimmer, Dublin, Ireland) that was attached to the participant’s earlobe. In accord with the SPA-*f*NIRS guidelines (Scholkmann et al., 2022), the recorded blood-pulse volume (frequency of sampling [fs] = 128 Hz) was regressed from the collected *f*NIRS signals to account for noncortical hemodynamic responses that represent potential confounds.

#### 3.3.4 fNIRS headset shift monitoring

Performing a motor task (e.g., cycling) can cause a shift in the position of the *f*NIRS headset. If a headset shift occurs during an experimental session, the exact source of recorded hemodynamic signals is rather difficult to determine. Thus, a motion capture technique (Qualisys MoCap, Götebord, Sweden) was used to detect shifts in the *f*NIRS headset within each experimental session. Specifically, one passive marker was taped to the participant’s right temple and two markers to the *f*NIRS headset.

To verify the occurrence of an *f*NIRS headset shift, the surface of the planar triangle connecting the 3D markers was computed over a 30-s timing window (a) at the beginning of the warm-up phase and (b) 30 s before volitional exhaustion (see Eq. 1; Guérin et al., 2021).

$$\vec{M_{0}M_{1}}\left(t\right)\cdot \vec{M_{0}M_{2}}\left(t\right)=\left(\begin{array}{c} x_{1}\left(t\right)\;-\;x_{0}\left(t\right)\\ y_{1}\left(t\right)\;-\;y_{0}\left(t\right)\\ z_{1}\left(t\right)\;-\;z_{0}\left(t\right) \end{array}\right)\cdot \left(\begin{array}{c} x_{2}\left(t\right)\;-\;x_{0}\left(t\right)\\ y_{2}\left(t\right)\;-\;y_{0}\left(t\right)\\ z_{2}\left(t\right)\;-\;z_{0}\left(t\right) \end{array}\right),$$ (1)

where 0 is the temple marker, 1 is the first headset marker, 2 is the second headset marker, and t is the time point. The percentage of variation between the two values was calculated. A shift in the *f*NIRS headset was identified when this value exceeded 15% (i.e., 10 mm). No participant’s entire data set was removed prior to further analyses due to a detected *f*NIRS headset shift (see Fig. 6).

#### 3.3.5 fNIRS data

The *f*NIRS neuroimaging technique was used to monitor the participants’ brain activity. This technique entails placing light source and detector optodes on the surface of the scalp. Adjacent sources and detectors of infrared light were ~3 cm apart. The depth of analysis into the cortex was 0.5–2.0 cm with the system that was used in the present study (FOIRE-3000/16; Shimadzu, Kyoto). The system’s light beam emanated from three lasers (class 1 M) at three wavelengths of 780, 805, and 830 nm. The equipment contained 16 light sources (multicomponent glass bundle fibers) and 16 detectors (multi-alkali photomultipliers detectors).

**Fig. 5.**
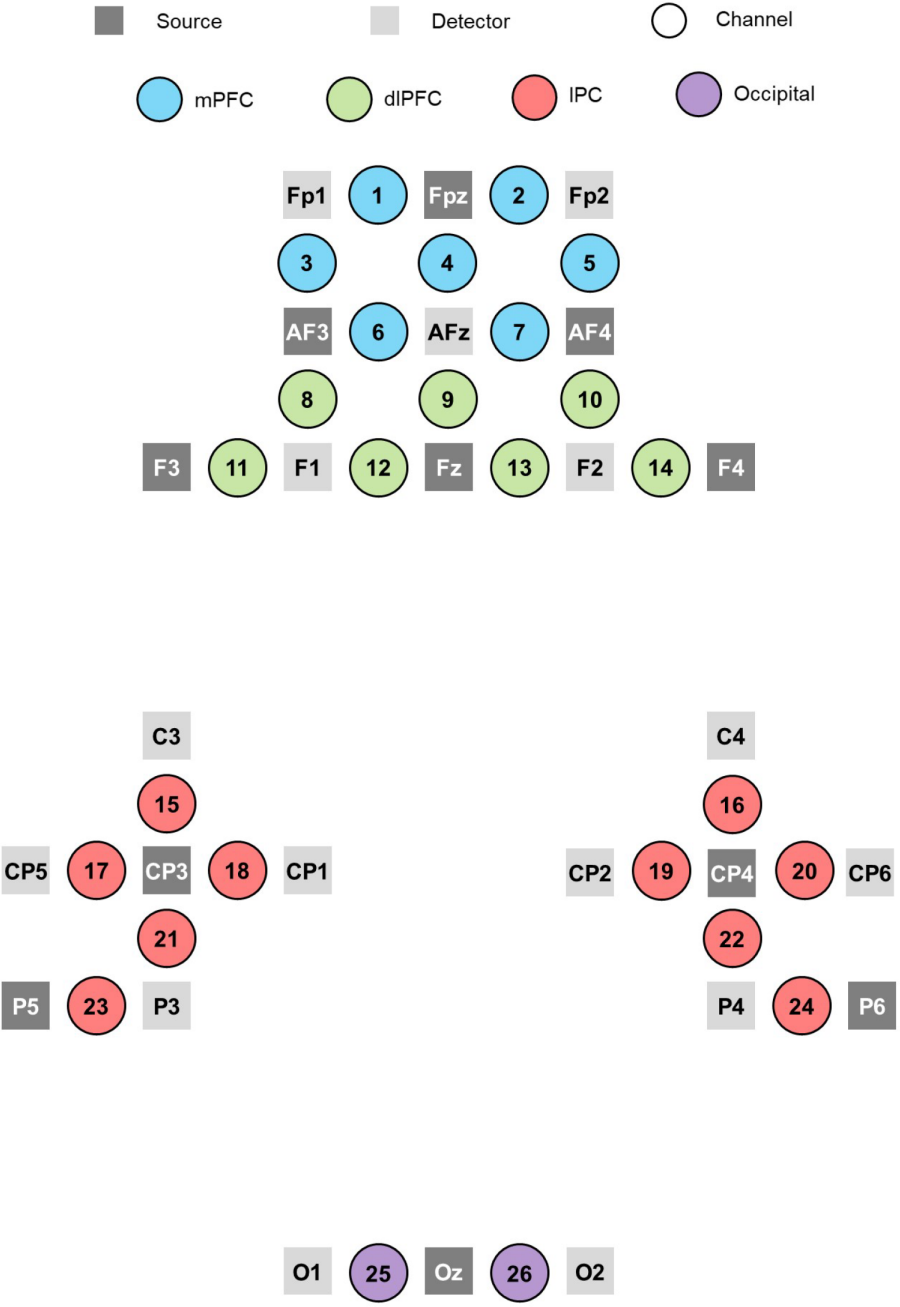
Diagrammatic representation of the *f*NIRS sources, detectors, and channel layout. *Note.* Adjacent sources and detectors were ~3 cm apart. mPFC = medial prefrontal cortex; dlPFC = dorsolateral prefrontal cortex; lPC = lateral parietal cortex.

The *f*NIRS headset holding the optodes was placed on the participant’s head in accord with the International 10–20 system guidelines for standard electrode positions (Jasper, 1958). In the present study, the brain regions of interest were the bilateral dlPFC (Brodmann areas [BAs] 9 and 46), medial prefrontal cortex (BAs 10 and 11), lateral parietal cortex (BA 39 and 40), and primary visual cortex (BA 17). Thus, a 26-channel model (11 sources and 15 detectors) was designed in order to cover the brain regions of interest (ROIs) over both the left and right hemispheres (see Fig. 5). The fOLD toolbox (*f*NIRS Optodes’ Location Decider; Morais et al., 2018) was used to guide the selection of optimal optode positioning with respect to the brain ROIs^3^ (see Supplementary File 3).

A system calibration was conducted at the beginning of each experimental session by means of automatic adjustment using LabNIRS to verify that all optodes were emitting correctly. In case that the amount of light detected was insufficient, the participant’s hair was pushed back beneath each problematic source–detector couple until data could be reliably collected. The sampling frequency was set at 10 Hz (i.e., temporal resolution of 100 ms).

To control for the quality of acquired *f*NIRS data, the power-spectral density was computed using Welch’s estimation method for each participant, session, and channel. The frequency corresponding to maximal peak in the 100–250 bpm range was detected in the power-spectral density of the raw *f*NIRS data (for a similar procedure, see Pinti et al., 2019). To guarantee that the identified frequency is the genuine heart rate frequency, it was compared with the heart rate measurements provided by the Polar system, with a tolerance threshold of 10 bpm (Guérin et al., 2021, 2023). A channel was excluded if heart rate frequency was not found in the *f*NIRS signals (see Fig. 4). In total, 9.4% of channels were excluded on this criterion. No participant’s entire data set was removed prior to further analyses on the basis that all channels pertaining to at least one ROI were excluded.

The presence of the heart pulse is a necessary but not sufficient condition to ensure the quality of *f*NIRS data (Pollonini et al., 2016). Thus, the QT-NIRS toolbox (Quality Testing of Near-Infrared Scans; Hernandez & Pollonini, 2020) was used to identify channels with poor optical coupling though the computation of the scalp-coupling index (cardiac filter = 2.5–4 Hz; time window = 5 s; λ = 805 and 830 nm). For a given participant and channel, *f*NIRS signals characterized by a scalp-coupling index <0.7 for at least 10% of the time segment of interest (i.e., 5%-above-VT1 phase) were removed prior to further analyses (0.18% of channels). As for the power-spectral density check, no participant’s entire data set was removed prior to further analyses on the basis that all channels pertaining to at least one ROI were excluded.

**Fig. 6.**
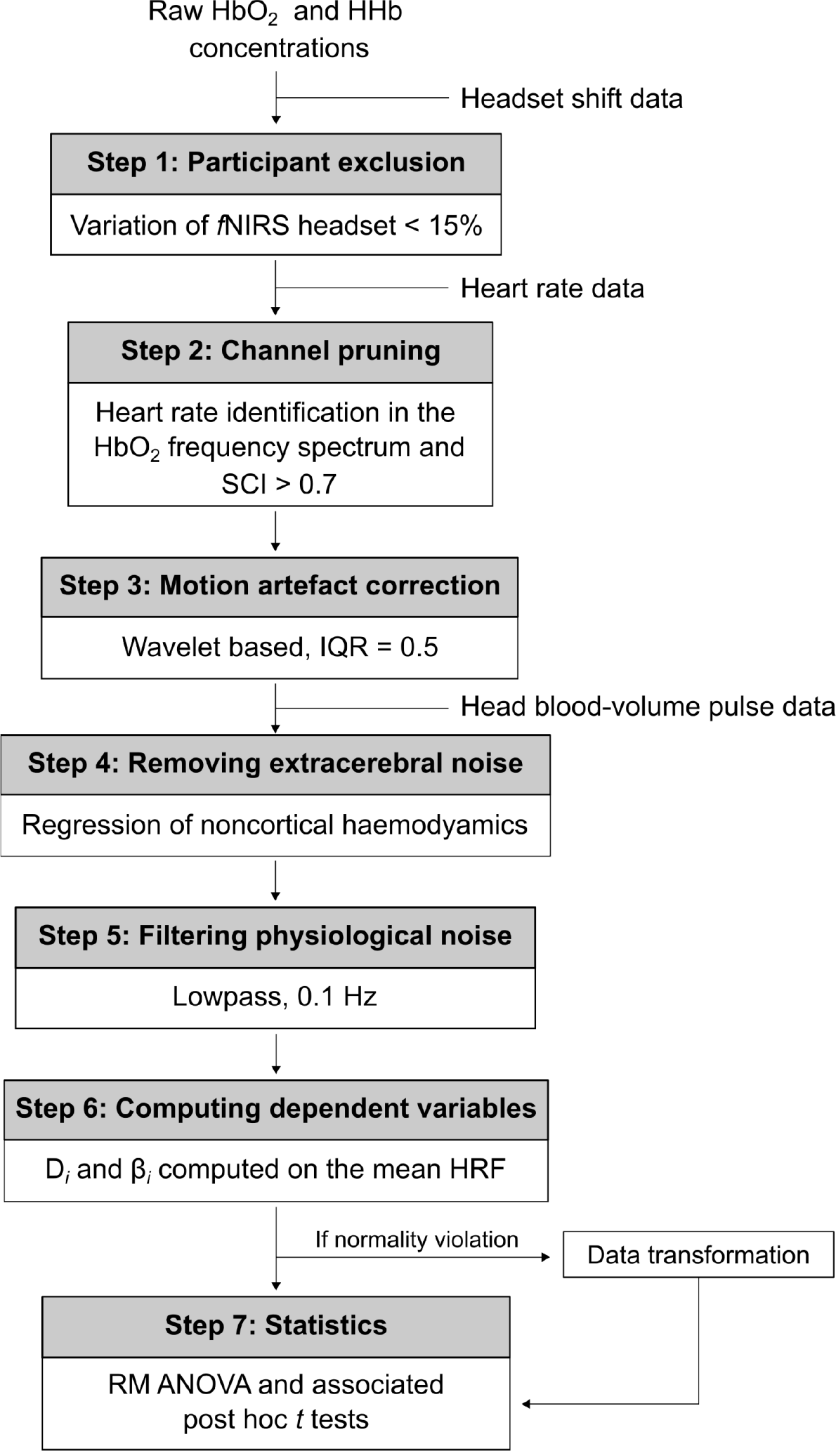
Processing pipeline of the *f*NIRS data. *Note. f*NIRS = functional near-infrared spectroscopy; SCI = scalp-coupling index; IQR = interquartile range; HRF = hemodynamic response function; RM ANOVA = repeated-measures analysis of variance.

Correction for motion artifacts was performed using wavelet filtering (interquartile range = 0.5) in Homer 3 (v1.58.0; Massachusetts General Hospital, Boston, MA). The motion-corrected data were visually inspected to ensure that the selected interquartile range value is well suited to the *f*NIRS data. For a given participant, visual inspection was performed to ensure that no motion artifacts were still visible (i.e., high-frequency spikes and/or baseline shifts) in the signal. To reject both cardiac and breathing rates along with parts of Mayer oscillations, a lowpass filter set at 0.1 Hz was applied (see Fig. 6).

**Fig. 7.**
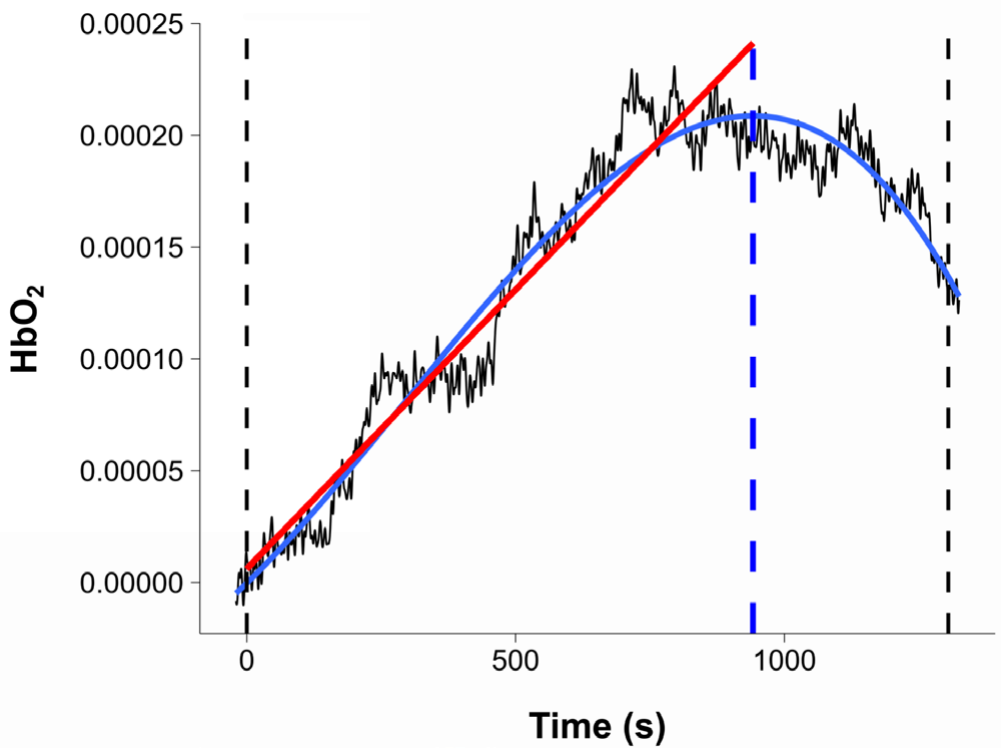
Computation of the dependent variables on orbitofrontal cortex *f*NIRS data. *Note.* These data were obtained from a pilot test. Dotted lines indicate the beginning and end of the 5%-above-VT1 phase. The polynomial regression is displayed in blue. The dotted blue line indicates the time point at which the maximal value of the polynomial regression was reached. The linear regression is displayed in red. Note that 0 on the *x* axis corresponds with the beginning of the 5%-above-VT1 phase. ${\text{HbO}}_{2}$ = oxygenated hemoglobin.

For each participant and condition, the *f*NIRS data between the beginning and end of the 5%-above-VT1 phase were extracted and referred to as a trial. The mean hemodynamic response function (HRF) was computed for each ROI (i.e., mPF, dlPFC, motor cortex, parietal cortex). For each trial *i*, a polynomial regression was fitted to the HRF. Thereafter, the decrease in cerebral oxygenation $D_{i}$ was defined as the time point at which the polynomial regression reaches its maximal value (see Fig. 7). To account for possible differences in exercise duration among participants, $D_{i}$ was not expressed in absolute time but rather as a percentage of the 5%-above-VT1 phase (e.g., if a participant exercised at 5% above VT1 for 10 min, and the maximal value of the polynomial regression was reached at 9 min, $D_{i}$ corresponded with 90%). To estimate the amplitude of changes in oxygenation during a trial, a linear regression was also fitted to each HRF from the beginning of the 5%-above-VT1 phase to $D_{i}$ (for a similar procedure, see Mandrick et al., 2013). The amount of cerebral oxygenation was identified by the slope coefficient of the linear regression, referred to as $\beta_{i}$ (see Fig. 7).

### 3.4 Statistical analyses

The statistical analyses were preregistered using the *f*NIRS preregistration template developed by Schroeder et al. (2023; see Supplementary File 4). The statistical analyses were performed using RStudio (v.1.2.5019). The raw data files and the associated data processing algorithms (pre-processing, statistics, and visualizations) are available here: https://doi.org/10.5281/zenodo.6261358.

#### 3.4.1 Data eligible for analysis

Participants characterized by a duration of the 5%-above-VT1 phase unusually long (i.e., >35 min) were removed prior to further statistical analyses (*n* = 2). Data were screened for univariate outliers using standardized scores (i.e., *z* scores). There were no participants with *z* scores >±3.29 , hence there was no need for any participant to be excluded.

#### 3.4.2 Classic null-hypothesis significance tests

Data from the questionnaires were analyzed by means of one-way repeated-measures (multivariate) analysis of variance (RM [M]ANOVA; audio condition [music, audiobook, control]). The cardiorespiratory and photoplethysmography data were also analyzed and reported in Supplementary Files 5 and 6, respectively. Because ${\text{HbO}}_{2}$ benefits from a better signal-to-noise ratio (see Gervain et al., 2011), only $D_{\text{HbO2}}$ and $\beta_{\text{HbO2}}$ were used to support or refute the hypotheses. Nonetheless, HHb indices were analyzed and the findings reported, in the interests of transparency (Yücel et al., 2025). $D_{\text{HbO2}}$ and $\beta_{\text{HbO2}}$ were analyzed for each ROI (see Suzuki et al., 2004) by means of RM ANOVAs for $H_{1}$–$H_{3}$. The critical statistical tests used to test hypotheses were the associated pairwise *t* tests from the post hoc analyses (see Table 1).

Normality was checked in each cell of the analysis using the Shapiro–Wilk test. Where normality was violated, a transformation was used in accord with the nature of the distribution curve (i.e., ordered quantile normalization; see Fig. 6). Where Mauchly’s test indicated violations of the sphericity assumption, Greenhouse–Geisser corrections were applied to the *F* test. Bonferroni adjustments pair-wise/multiple comparisons were used, as appropriate to identify where differences lie. The significance level was set at *p* < .020 for all analyses. Partial eta squared and Cohen’s *d* effect sizes are reported alongside each inferential analysis.

#### 3.4.3 Outcome-neutral validation tests

A negative control condition was included by placing two additional channels over the occipital brain region (Broadmann’s area 17). This region is involved primarily in visual perception and so its activation should not differ in response to the experimental conditions. To confirm that similar hemodynamic responses of the primary visual cortex were observed regardless of the audio condition ($H_{4}$), two one-sided tests (TOSTs) were used (Lakens et al., 2018). In this procedure, the results of both *t* tests needed to reach significance in order for equivalence to be claimed. Statistically nonsignificant differences provide a means by which to confirm that observed mPFC, dlPFC, and parietal differences are related to the audio manipulations. TOSTs were computed using the TOSTER R package for paired-samples *t* tests (Lakens, 2017).

## 4 Results

Cardiorespiratory and photoplethysmography results are reported in Supplementary Files 5 and 6, respectively. HHb results are reported in Supplementary File 7.

### 4.1 Data screening and diagnostics

Data screening indicated that there were six univariate outliers, associated with $\beta_{{\text{HbO}}_{2},\text{mPFC}}$ (*k* = 1), $\beta_{{\text{HbO}}_{2},\text{dlPFC}}$ (*k* = 1), $\beta_{{\text{HbO}}_{2},\text{lPC}}$ (*k* = 2), $\beta_{{\text{HbO}}_{2},\text{occipital}}$ (*k* = 1), and exercise duration (*k* = 1) measures. These were adjusted using a winsorization procedure (Sullivan et al., 2021) until they came within the range -3.29 < z > + 3.29 (see Tabachnick & Fidell, 2018).

Normality tests indicated that affective valence (*p* < .001), affective arousal (*p* < .001), perceived exertion (*p* = .025), attentional focus (*p* < .001), remembered pleasure (*p* < .001), and exercise duration (*p* < .001) exhibited instances of non-normality. An ordered quantile normalization transformation (Peterson & Cavanaugh, 2020) was applied to affective valence, perceived exertion, remembered pleasure, and exercise duration to remedy this; affective arousal and attentional focus were resistant to the transformation. Thus, the nonparametric Friedman rank sum test was employed for these two dependent variables. The normality assumption was also not met for $D_{{\text{HbO}}_{2}}$ (*p* < .001) in each of the two brain regions of interest, and proved resistant to various transformations (e.g., rank-based inverse normal transformation, square-root, reflect, and log/square root, Yeo–Johnson transformation). Accordingly, the nonparametric Friedman rank sum test was employed to test $H_{1}$. Finally, normality tests indicated that $\beta_{{\text{HbO}}_{2}}$—for each of the three brain regions of interest—exhibited instances of non-normality (*p* < .001), and ordered quantile normalization transformations were applied to remedy this.

### 4.2 *f*NIRS

#### 4.2.1 Decrease in cerebral oxygenation

**Fig. 8.**
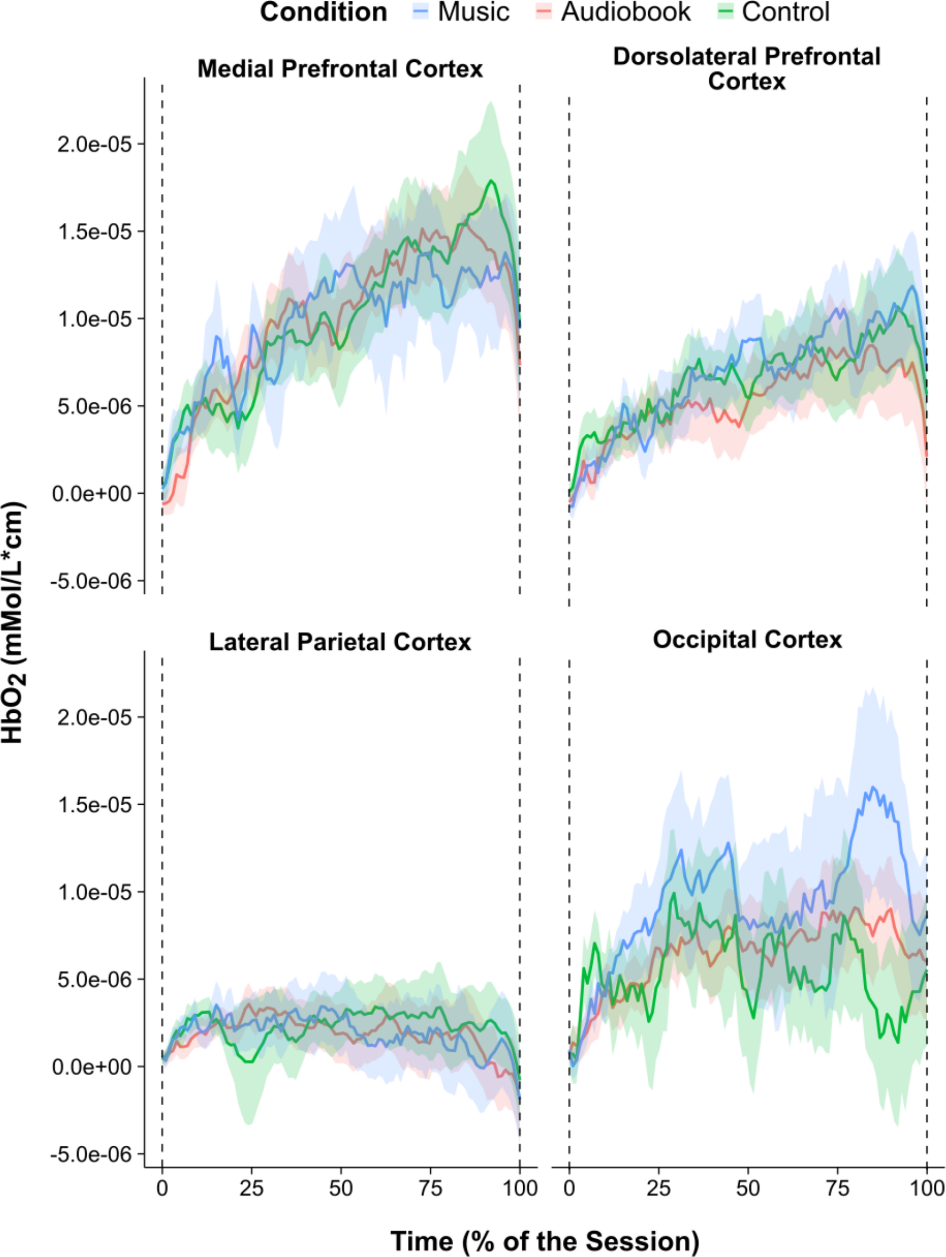
Mean hemodynamic response functions. *Note.* Mean hemodynamic response function for each condition and brain region of interest. The ribbons represent the mean ± one standard error.

The Friedman test computed for $D_{{\text{HbO}}_{2}}$ in the mPFC showed no significant main effect of condition, $\chi^{2}$ (2) = 3.18, *p* = .203, *W* = .04. The Friedman test performed on $D_{{\text{HbO}}_{2}}$ in the dlPFC was also nonsignificant, $\chi^{2}$ (2) = 0.65, *p* = .723, *W* = .01. Overall, it was evident that the delay in cerebral oxygenation decrease before volitional exhaustion was not affected by condition (see Fig. 8). In this regard, the mean decrease in cerebral oxygenation in the mPFC occurred at 78.4%, 77.6%, and 92.6% of the 5%-above-VT phase in the music, audiobook, and control conditions, respectively. In the dlPFC, the mean decrease in cerebral oxygenation was at 77.4% of the 5%-above-VT phase in the audiobook condition; in the other two conditions, the mean polynomial function was monotonic (i.e., increased on a linear trajectory).

#### 4.2.2 Amplitude of activation

The one-way RM ANOVA on $\beta_{{\text{HbO}}_{2}}$ in the mPFC showed no significant main of condition, *F*(2, 38) = 1.95, *p* = .156, $\eta_{\text{p}}^{2}$ = .09. The one-way RM ANOVA on $\beta_{{\text{HbO}}_{2}}$ in the dlPFC also showed no significant main of condition, *F*(2, 42) = 0.74, *p* = .485, $\eta_{\text{p}}^{2}$ = .03. The one-way RM ANOVA on $\beta_{{\text{HbO}}_{2}}$ in the lateral parietal cortex was also nonsignificant, *F*(2, 70) = 0.07, *p* = 0.931, $\eta_{\text{p}}^{2}$ < .01. Overall, the results indicated that activation of the mPFC, dlPFC, and lateral parietal cortex was not influenced by condition (see Fig. 8).

#### 4.2.3 Negative control

The TOST procedure applied to $\beta_{{\text{HbO}}_{2}}$ in the occipital cortex was significant for music vs. audiobook condition (*t*[35] = 3.63, *p* < .001, Hedges’ *g* = 0.01), music vs. control condition (*t*[35] = 2.40, *p* = .011, Hedges’ *g* = -0.22), and audiobook vs. control condition (*t*[35] = -2.32, *p* = .013, Hedges’ *g* = .23). Overall, the results indicated that activity in the occipital cortex was similar across conditions.

### 4.3 In-task measures

#### 4.3.1 Core affect

**Fig. 9.**
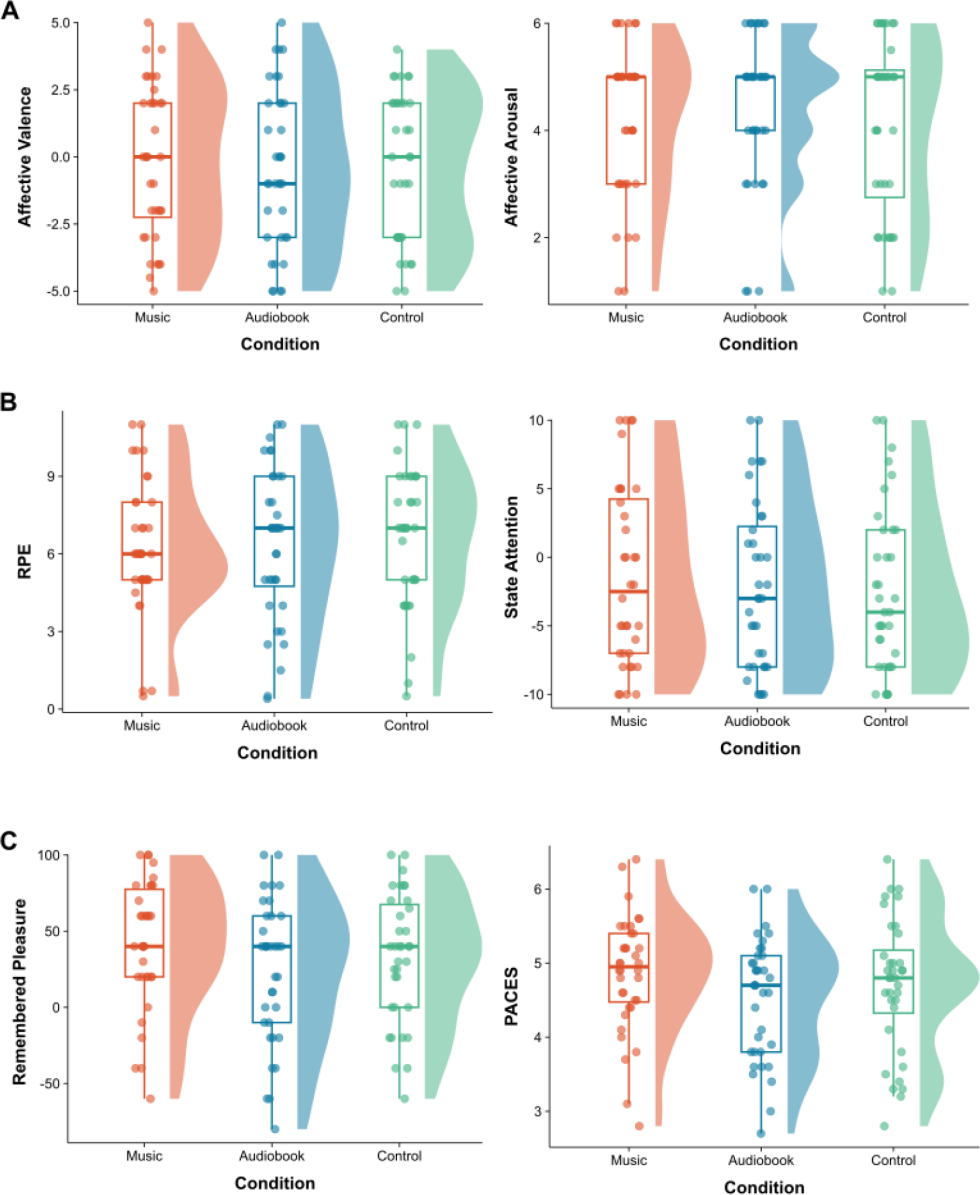
In-task and post-task measures. *Note.* (A) Core affect. (B) In-task measures of perceived exertion and attentional focus. (C) Post-task measures of remembered pleasure and physical activity enjoyment. RPE = rating of perceived exertion; PACES = Physical Activity Enjoyment Scale. Box plots and probability density functions are displayed for each condition, using the non-normalized data. Each dot represents an individual participant.

The one-way RM ANOVA showed no significant main effect of condition for affective valence, *F*(2, 70) = 1.13, *p* = .329, $\eta_{\text{p}}^{2}$ = .03. The Friedman rank sum test performed on affective arousal was also nonsignificant, $\chi^{2}$ (2) = 0.29, *p* = .864, *W* < .01 (see Fig. 9A). Overall, the results indicated that core affect was not influenced by condition.

#### 4.3.2 Perceived exertion and state attention

The one-way RM ANOVA on perceived exertion showed no significant main effect of condition, *F*(2, 70) = 2.32, *p* = .106, $\eta_{\text{p}}^{2}$ = .06. The Friedman rank sum test performed on attentional focus was also nonsignificant, $\chi^{2}$ (2) = 0.64, *p* = .725, *W* < .01. Overall, the results indicated that perceived exertion and attentional focus were not influenced by condition (see Fig. 9B).

### 4.4 Post-task measures

The one-way RM ANOVA on remembered pleasure showed no significant main effect of condition, *F*(2, 66) = 2.51, *p* = .089, $\eta_{\text{p}}^{2}$ = .07, as did the one-way RM ANOVA on physical activity enjoyment, *F*(1.65, 57.85) = 3.55, *p* = .043, $\eta_{\text{p}}^{2}$ = .09. Overall, the results indicated that remembered pleasure and physical activity enjoyment were not influenced by condition (see Fig. 9C).

### 4.5 Headset position tracker

The variation in the position of the *f*NIRS headset did not exceed the 15% threshold for any of the participants (*M* = 2.47%, *SD* = 2.48, min = 0.03%, max = 14.95%). This served to confirm the absence of *f*NIRS headset shift from the beginning to the end of the experiment.

### 4.6 Exploratory analyses

#### 4.6.1 Volitional exhaustion

**Fig. 10.**
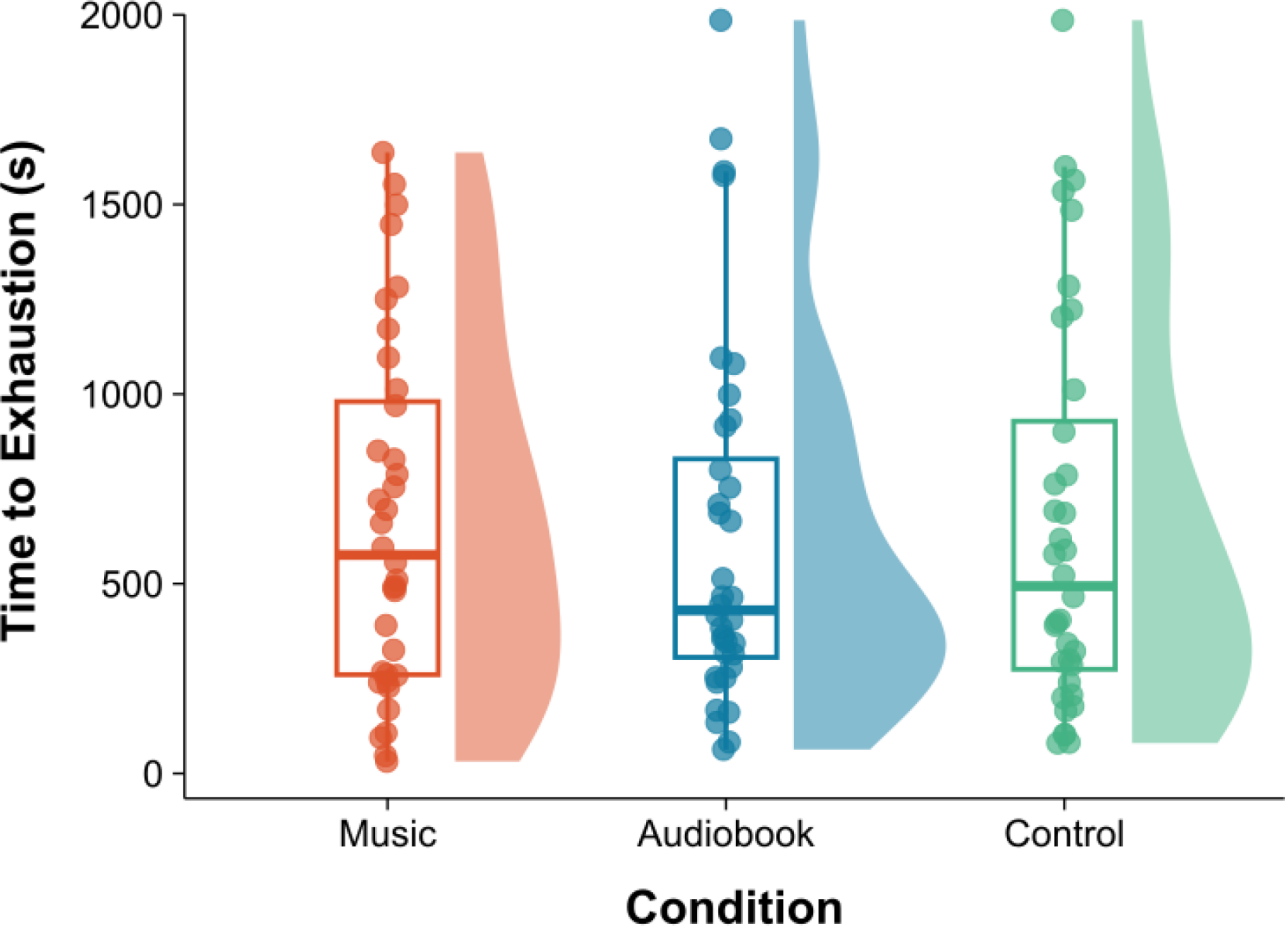
Time to volitional exhaustion. *Note.* Box plots and probability density functions are displayed for each condition, using the non-normalized data with the outlier removed. Each dot represents an individual participant.

One outlier was corrected using a winsorization procedure (see Sullivan et al., 2021). The normality assumption was not met for the exercise duration (*p* < .001) and an ordered quantile normalization transformation (Peterson & Cavanaugh, 2020) was applied to remedy this. A one-way RM ANOVA was performed on exercise duration during the 5%-above-VT1 phase, and there was no significant effect of condition, *F*(2, 70) = 0.28, *p* = .753, $\eta_{\text{p}}^{2}$ = .01 (see Fig. 10).

#### 4.6.2 Delta value

Considering the pattern displayed in Figure 9, it could be that it is actually Hypothesis B of Karageorghis et al. (2017) that is verified, at least at the level of the mPFC. To test this, the delta value between the baseline and the end of the 5%-above-VT-phase, averaged over a 5 s window, was computed for the mPFC. Outliers (*k* = 3) were corrected using a winsorization procedure (Sullivan et al., 2021). The normality assumption was not met for the delta value (*p* < .001) and proved resistant to various transformations. Consequently, the nonparametric Friedman rank sum test was employed, which proved nonsignificant, $\chi^{2}$ (2) = 1.35, *p* = .510, *W* = .02.

#### 4.6.3 Correlation between core affect and $\text{Hb}{\text{O}}_{\text{2}}$

The correlation coefficients between core affect (i.e., affective valence and arousal) and the various ${\text{HbO}}_{2}$ indices (i.e., $D_{{\text{HbO}}_{2}}$, $\beta_{{\text{HbO}}_{2}}$, and delta) in the mPFC and dlPFC were computed using Pearson’s product–moment correlation. The affective valence–$D_{{\text{HbO}}_{2}}$ value and affective arousal–$D_{{\text{HbO}}_{2}}$ value correlations were nonsignificant for both the mPFC ($r_{\text{valence}}$ = -.16, *p* = .104, 95% confidence interval [CI] = [-.34 to .03]; $r_{\text{arousal}}$ = .07, *p* = .444; 95% CI = [-.12 to .26]) and the dlPFC ($r_{\text{valence}}$ = -.09, *p* = .347, 95% CI = [-.28 to .10]; $r_{\text{arousal}}$ = .10, *p* = .317, 95% CI = [-.09 to .28]). The affective valence–$\beta_{{\text{HbO}}_{2}}$ value and affective arousal–$\beta_{{\text{HbO}}_{2}}$ value correlations were nonsignificant for both the mPFC ($r_{\text{valence}}$ = .04, *p* = .713, 95% confidence interval [CI] = [-.17 to .24]; $r_{\text{arousal}}$ = -.07, *p* = .524; 95% CI = [-.27 to .14]) and the dlPFC ($r_{\text{valence}}$ = .05, *p* = .668, 95% CI = [-.16 to .25]; $r_{\text{arousal}}$ = -.07, *p* = .545, 95% CI = [-.27 to .15]).

A correlation between affective valence and the delta value was significant for the mPFC (r = -.26, *p* = .006, 95% confidence interval [CI] = [-.43 to -.08]), but not between affective arousal and the delta value r = .19, *p* = .047; 95% CI = [.01 to .37]). In addition, the affective valence–delta value and affective arousal–delta value correlations were significant for the dlPFC ($r_{\text{valence}}$ = -.22, *p* = .020, 95% CI = [-.39 to -.03]; $r_{\text{arousal}}$ = .23, *p* = .017, 95% CI = [.04 to .40]). Overall, the results indicated a small negative correlation between affective valence scores and activation in the mPFC and dlPFC (i.e., the higher the affective valence, the lower the activation), along with a small positive correlation between affective arousal scores and activation of the dlPFC (i.e., the higher the affective arousal, the higher the activation).

#### 4.6.4 Modeling of the fNIRS response

The use of two linear regressions has been reported in the literature as an effective approach for characterizing the time point at which cerebral oxygenation begins to decrease (see e.g., Oussaidene et al., 2015). Accordingly, the registered polynomial regression was formally compared with a two-segment linear regression model. Specifically, a standard linear model was first fitted to the data. The breakpoint (i.e., the time at which the slope of ${\text{HbO}}_{2}$ changes) was then estimated using the “segmented” R package and used as a model parameter. Two connected linear functions were subsequently fitted to the data: one for the interval preceding the breakpoint and another for the interval following it. Finally, the Akaike information criterion (AIC) and Bayesian information criterion (BIC) were computed to enable a comparison between the two modeling approaches (i.e., polynomial regression vs. two-segment linear regression) in terms of model fit (Cavanaugh & Neath, 2019).

A paired-samples *t* test was conducted on both the AIC and BIC. The *t* test was significant for both AIC, *t*[215] = 3.16, *p* = .002, Cohen’s *d* = 0.21, and BIC, *t*[215] = 3.16, *p* = .002, Cohen’s *d* = 0.21. Thus, model comparison showed evidence in favor of the two-segment linear regression model ($M_{AIC}$ = -144188.0, $M_{BIC}$ = -144155.7) when compared with the polynomial model ($M_{AIC}$ = -143938.9, $M_{BIC}$ = -143906.6), indicating that the *f*NIRS time course is better characterized by two linear phases separated by a breakpoint.

The $D_{{\text{HbO}}_{2}}$ values for the mPFC and dlPFC computed using the two-segment linear regression model were analyzed by means of one-way RM ANOVA. The RM ANOVA computed for $D_{{\text{HbO}}_{2}}$ in the mPFC showed no significant main effect of condition, *F*(2, 70) = 0.87, *p* = .417, $\eta_{\text{p}}^{2}$ = .02. The RM ANOVA performed on $D_{{\text{HbO}}_{2}}$ in the dlPFC was similarly nonsignificant, *F*(1.69, 59.14) = 2.19, *p* = .129, $\eta_{\text{p}}^{2}$ = .06. Collectively, this provides evidence that the decrease in cerebral oxygenation was not affected by condition, irrespective of the modeling approach used to determine the onset point of the decrease.

## 5 Discussion

The main purpose of the present study was to assess the effects of motivational asynchronous music on the cerebral oxygenation time series during a constant-rate cycle ergometer task to volitional exhaustion, commencing at 5% above VT1. Brain oxygenation was recorded by means of a continuous-wave *f*NIRS system, and a number of self-report psychological (e.g., core affect) and psychophysical (e.g., RPE) in-task measures were taken at regular intervals. Post-task measures of physical activity enjoyment and remembered pleasure were taken at the end of each experimental session. Collectively, these in-task and post-task subjective measures enabled the research team to ascertain how the subjective experience of exhaustive exercise tessellated with the objective data derived from the *f*NIRS system.

The hypothesis stating that the decrease in prefrontal oxygenation would be observed earlier in the silence and audiobook conditions when compared with asynchronous music ($H_{1}$) was not accepted (see Fig. 8). The hypothesis that exercising with music would lead to less prefrontal activation when compared with exercising in silence or with an audiobook ($H_{2}$) was also not accepted (see Fig. 8). Moreover, the hypothesis that exercising in silence or with an audiobook would lead to less parietal activation when compared with exercising with music ($H_{3}$) was not accepted (see Fig. 8). The negative control hypothesis ($H_{4}$) stating that occipital cortex activation would not differ among experimental conditions was accepted (see Fig. 8).

### 5.1 Effects of music on cerebral hemodynamics and exercise endurance

Contrary to expectations, the cerebral oximetry did not show any significant (*p* < .020) effect of asynchronous music either in prefrontal or parietal oxygenation. There is, however, a trend evident that is somewhat in accord with Karageorghis et al.’s (2017) Hypothesis B (see Fig. 1); specifically, that music delays the increase in prefrontal oxygenation due to a reallocation of attention (see Medial Prefrontal Cortex in Fig. 8). This trend emerges only in the last 25% of the cycle ergometer protocol—specifically, ~80%. Besides a moderate effect size of the omnibus test for $D_{{\text{HbO}}_{2}}$ (Kendall’s *W* = .03), the variability associated with the ${\text{HbO}}_{2}$ trace for each of the three conditions (see ribbons in Fig. 8) clearly inhibits this trend from reaching statistical significance. Moreover, our initial intention was to use a parametric approach (i.e., RM ANOVA) to test this hypothesis. Nonetheless, due to ${\text{HbO}}_{2}$ being resistant to various transformations to remedy moderate negative skewness in each of the two brain regions of interest, we computed a nonparametric Friedman rank sum test.

It is worthwhile considering the probable reasons for both the variability and non-normality in the *f*NIRS data, as these will be important touchpoints for future researchers with an interest in exercise-related hemodynamics. The varying lengths of time that participants endured the 5% above VT cycle ergometer task are a primary source of error (see Fig. 10), an issue commonly encountered in time-to-exhaustion protocols (Bossi et al., 2024; Karsten et al., 2018), albeit this was mitigated somewhat by taking the percentage of the 5%-above-VT1 phase rather than the absolute time. Coupled with this is the heterogeneous background of participants in terms of sport and physical activity (see Supplementary File 8). High variability cannot, however, be attributed to sex differences, as oxygenation curves were similar across the sexes (see Supplementary File 9). With reference to instances of non-normality, this was negative skewness in all cases, which can be attributed to the decrease in cerebral oxygenation timing being congregated toward the high end of the distribution (i.e., the time of decrease in oxygenation is quite high; see Supplementary File 9). This is due to the fact that in some participants, the hemodynamic response function displays a pattern of linear increase in activation (i.e., increase of ${\text{HbO}}_{2}$ throughout, without any decline; see Zohdi et al., 2021), with the maximal value of the polynomial regression thus reached at the very end of the 5% above VT cycle ergometer segment (see Supplementary File 10).

Examining the present findings in light of closely related findings, it is clear that direct comparisons are limited by the fact that no previous study involving an auditory or audiovisual manipulation adopted an exercise-to-exhaustion-type protocol (Jones & Ekkekakis, 2019; Suwabe et al., 2021; Wang et al., 2025). In this regard, previous studies report enhancements in affective valence and dissociation as a consequence of the manipulation of exteroceptive cues, and the benefits are particularly noticeable in the case of low-active adults (Jones & Ekkekakis, 2019). Moreover, studies have shown that such manipulations can have a discernible effect on cerebral hemodynamics—represented by higher oxygenation in the right dlPFC—especially when audiovisual stimuli are self-selected (Wang et al., 2025). Neither the exercise tasks employed in previous studies that entailed administration of audiovisual stimuli nor the task employed in the present study elicited clear and complete dlPFC oxygenation–deoxygenation curves as depicted in the Karageorghis et al. (2017) hypotheses (see Fig. 1).

What is noticeable in the present data is a tentative trend toward less neural stimulation in the mPFC coupled with a longer time to exhaustion during the asynchronous music condition (see Figs. 8 and 10). It is important to stress that neither of these trends was associated with statistical significance; nonetheless, given that a longer time spent exercising is likely to impose greater cerebral and cardiovascular demands on the organism (Smith & Ainslie, 2017), this was not reflected in the oxygenation curve for the music condition. What is equally interesting is that in the mPFC, the audiobook condition engendered the greatest neural stimulation coupled with the shortest time to exhaustion (see Fig. 8). Given the aforementioned variability in the present data and attendant lack of significance, these emerging patterns are certainly worthy of further examination.

### 5.2 In-task self-report measures

The auditory manipulations did not have any significant (*p* < .020) effect on in-task self-report measures (see Fig. 9A, B). Similar to the *f*NIRS data, there is considerable variability in these data when viewed through the lens of closely related work (e.g., Hutchinson & Karageorghis, 2013; Jones & Wheat, 2023), which could be a reflection of the varying cardiorespiratory fitness and motivation levels of the participants (see Supplementary File 8). Nonetheless, the fact that the exercise protocol was conducted at 5% above VT meant that the auditory stimuli were less likely to render the exercise experience more pleasant when compared with intensities at VT or below VT (e.g., Bird et al., 2016; Jones & Ekkekakis, 2019). Beyond VT, the predominance of interoceptive cues means that exteroceptive cues, such as motivational music, are rendered less salient in moderating exercise-related feelings and perceptions (Jones et al., 2024; Rejeski, 1985; Tenenbaum, 2001). Few previous studies, however, have been conducted with participants wearing an *f*NIRS headset (e.g., Jones & Ekkekakis, 2019; Suwabe et al., 2021) and there is, therefore, a possibility that this aspect also restricted the salience of the music-based intervention (i.e., wearing the headset creates some discomfort and/or anxiety). Moreover, previous studies employed a lower exercise intensity than the present study (e.g., Suwabe et al., 2021; Wang et al., 2025).

In a 15-min recumbent cycle protocol at VT, Jones and Ekkekakis (2019) showed that higher levels of right dlPFC oxygenation were associated with lower scores for affective valence in a high exercise-intensity preference group. The present findings showed weak and nonsignificant (*p* < .020) correlations, with the exception of affective valence and the delta value for the dlPFC (*r* = -.25, *p* = .009). The equivalent Jones and Ekkekakis (2019) correlation was, however, nonsignificant. Notably, Wang et al. (2025) reported rather similar correlations to those reported herein, as their *r* values for right dlPFC and left dlPFC were weak (.21 and -.17, respectively) and nonsignificant. Collectively, the extant evidence shows that there is a weak-to-negligible correlation between dlPFC oxygenation and affective valence scores in exercise-related protocols.

### 5.3 Post-task self-report measures

The auditory manipulations did not have any significant (*p* < .020) effect on post-task self-report measures (see Fig. 9C). The reasons for which the music intervention did not influence remembered pleasure and exercise enjoyment are rather similar to those detailed in the previous subsection regarding in-task measures. However, the in-task measures were taken at 2.5-min intervals, whereas the two post-task measures were administered immediately upon cessation of warm down. With reference to the peak–end rule (Kahneman et al., 1993), following a bout of exercise conducted to the point of volitional exhaustion, it is unsurprising that the auditory interventions did not modulate the post-task psychological responses (i.e., a gestalt evaluation). The experience of wearing an *f*NIRS headset (inc. the 20-min preparatory phase of manipulating the participant’s hair) may have negatively tinged—in affective terms at least—the entire exercise bout.

### 5.4 Strengths and weaknesses

The present study employed a rigorous music selection procedure that did not involve engaging the experimental participants themselves, which has the potential to introduce a source of bias (i.e., expectancy effect; see Karageorghis, 2020). The exercise intensity was set with reference to a physiological event (i.e., VT1, defined using the heart rate variability index of RMSSD; see Supplementary File 2) and not a percentage of each participant’s maximal heart rate, as is the case in many related studies (e.g., Wang et al., 2025; Yang et al., 2022). Accordingly, the starting exercise intensity across participants was fully standardized. Environmental conditions within the laboratory (i.e., temperature) were also standardized by means of a climate-control system. An outcome-neutral validation test (or “sanity check”) was included that entailed monitoring cerebral hemodynamics in the occipital cortex (see Fig. 8). Using a TOST procedure, the test indicated that activity in this brain region was similar across conditions.

We used a photoplethysmograph sensor to monitor and remove noncortical hemodynamic responses—specifically perfusion of blood to the dermis and subcutaneous tissue of the skin (see Supplementary File 6). Such an approach serves to enhance the signal-to-noise ratio in *f*NIRS data (Yücel et al., 2021) and is in accordance with current recommendations in the field (e.g., Scholkmann et al., 2022; von Lühmann et al., 2020). Although this method cannot eliminate *all* physiological noise, it can reduce systemic noise by ~30% (Sutoko et al., 2019). The control for extracerebral noise showed low median correlations between the hemodynamic response and photoplethysmograph signal for each designated *f*NIRS index ($Mdn_{{\text{HbO}}_{2}}$ = .148; $Mdn_{\text{HHb}}$ = .107; see Supplementary File 6). We can deduce from this that variations in oxygen levels in the recorded hemodynamic responses were affected only to a small degree by the non-neural, systemic physiological activity.

The study was not without some limitations and chief among these was the use of a fixed intensity at 5% above VT1, which resulted in differing task completion times across participants (i.e., range: 31.8–1,985.7 s; $M_{\text{Music}}$ = 666.9 ± 464.1 s, $M_{\text{Audiobook}}$ = 617.4 ± 482.2 s, $M_{\text{Control}}$ = 655.2 ± 517.6 s). In addition, the fixed exercise intensity was 5% above VT1, a metabolic marker that is lower that VT2—the threshold typically used in this type of exercise protocol (see Ochi et al., 2018; Rupp & Perrey, 2008). The protocol employed relied upon the phenomenon of physiological drift (confirmed with heart rate and RPE measurements; see Supplementary Files 11 and 12) for participants to reach the point of volitional exhaustion, but because there was considerable variation in the drift period across participants, this introduced a source of between-subject error (see e.g., Fig. 10). The variability is evident across the entire suite of dependent variables (i.e., subjective and objective). To further illustrate this point, visualization of the time series for systemic physiological signals (i.e., heart and respiratory rates) is available in Supplementary File 12.

A possible alternative approach would be to employ an incremental protocol (Karapetian et al., 2008), but the research team were mindful of this inhibiting the testing of $H_{1}$–$H_{3}$ due to the sudden cessation of exercise following an imposed increase in intensity. The exercise protocol employed was deemed to better reflect how people exercise “in the wild”, with the upshot that it introduced greater variability in task completion time. Another limitation is that the effect sizes used for the power analysis were derived from published, nonregistered studies, which likely led to substantial overestimation of effect sizes (see Schäfer & Schwarz, 2019; van den Akker et al., 2024): a phenomenon that might be attributed to publication bias (Algermissen & Mehler, 2018; Lakens et al., 2024). This relates to the fact that the chances for null findings with registered reports are significantly larger (Allen & Mehler, 2019; Scheel et al., 2021).

Determining VT1 based on an indirect cardiac metric (i.e., heart rate variability index of RMSSD) is a further source of bias to consider. Moreover, data collection took place over one calendar year and so different participants were tested in different seasons, which can have a small bearing on their affective responses (Winthorst et al., 2020), and thus contribute an additional source of between-subject error. We aimed to recruit participants in the age range 18–35 years, but there was a large gap in age from a group in the range 19–25 years up to a single participant at 34 years. We did closely scrutinize the responses of the 34-year-old through singling them out (via color coding) in boxplots for each dependent variable, but they did not differ from their younger counterparts on any dependent variable.

### 5.5 Implications for practice and directions for future research

Gaining a fuller understanding of how cerebral hemodynamics are affected by auditory stimuli can serve to refine strategies that are intended to promote exercise behaviors (Wang et al., 2025). The findings of the present study hint toward there being some credibility in the hypothesis of Karageorghis et al. (2017) that the presence of pleasant auditory stimuli can serve to reduce the “level of unpleasantness” experienced during exercise. Added to this, the music had a mild ergogenic effect (see Fig. 10) that although did not reach statistical significance (i.e., *p* > .02) is notable from a public health perspective.

The exercise intensity selected for the present study was relatively high (see Supplementary Files 11 and 12 for the time series of heart rate and RPE data) and so to reap the benefits of music in upregulating affective responses while assuaging RPE, practitioners need to consider its application at low-to-moderate exercise intensities (Karageorghis, 2020; Kuan et al., 2026). It has been shown in previous work that the efficacy range of music is higher in this range (e.g., Hutchinson & Karageorghis, 2013; Moore et al., 2024). Certainly, from the present intensity of 5% above VT and beyond that, the evidence for benefits to affective responses is sketchy, and the benefits in terms of assuaging RPE are negligible (see Marques et al., 2024; Terry et al., 2020). In an applied setting, self-selection of music is likely to magnify any benefits in terms of affective and perceptual responses (e.g., Hutchinson et al., 2018; Wang et al., 2025).

The present study illuminates a path toward a number of follow-on studies that would shed further light on cerebral hemodynamics during exercise. Predicated on the hemispheric asymmetry hypothesis (Hellige, 1995), the left and right hemispheres of the brain could be examined using *f*NIRS to gauge whether there are differential responses. Specifically, the prediction is that the left side dominates in processing positive emotional experiences and the right side the converse. Albeit the hypothesis has recently been tested in the context of submaximal exercise (Wang et al., 2025), it has not been tested with severe intensity or exhaustive exercise, which would, in fact, make for a more robust test.

There were several sources of between-subject error identified in the present study. For example, participants’ varying fitness/endurance levels and testing across all four seasons. To better characterize the oxygenation curve and assess the influence of exteroceptive cues such as asynchronous music, future researchers might consider recruiting a more homogeneous sample than that recruited for the present study. It is duly acknowledged that this presents certain practical challenges, but the pattern of change in cerebral hemodynamics in exercise-related studies is so nuanced that the use of a heterogeneous sample can serve to obfuscate important trends. Gaining better understanding of these mechanisms is important from the perspective of being able to render the exercise experience more pleasant and, by extension, promoting adherence (Ekkekakis, 2020).

## 6 Conclusion

The main purpose of this study was to assess the effects of pleasurable auditory stimuli (i.e., motivational asynchronous music) on the cerebral oxygenation curve during an exhaustive cycle ergometer task. The experimental conditions did not have any significant (*p* < .020) influence on cerebral hemodynamics, exercise endurance, or a range of psychological and psychophysical measures. There was a nonsignificant trend evident toward less stimulation in the mPFC and greater exercise endurance in the music condition (see Figs. 8 and 10). This trend shows that there is considerable scope to re-examine how auditory stimuli influence exercise endurance and the underlying neurophysiological mechanisms. A weakness of the present study was the relatively high between-subject error that resulted, in particular, from participants’ differing levels of cardiorespiratory fitness/endurance (see Supplementary File 8). This could be overcome in the future through testing a more homogenous sample. Alternatively, an incremental protocol could be used, but the increments would need to be relatively small in order that there is sufficient scope for the cerebral oxygenation curve to emerge.

## Supplementary Material

Supplementary Material 1

Supplementary Material 2

Supplementary Material 3

Supplementary Material 4

Supplementary Material 5

Supplementary Material 6

Supplementary Material 7

Supplementary Material 8

Supplementary Material 9

Supplementary Material 10

Supplementary Material 11

Supplementary Material 12

## Data Availability

The study data and materials are shared openly as part of the publication of the article. All anonymized raw and processed data supporting the reported analyses, along with the code used for preprocessing and analyses, are available on a public Zenodo repository (https://doi.org/10.5281/zenodo.6261358). Methodological details pertaining to the present study were preregistered using the *f*NIRS preregistration template developed by Schroeder et al. (2023; see Supplementary File 4).

## Footnotes

1 The effect sizes used for the sample size computation and SESOI are two distinct entities. More precisely, the sample size computation serves to ensure that the study is appropriately powered (i.e., good probability that the statistical test will detect an effect that actually exists), while the SESOI serves to ensure that a true effect exists (for further details, see Sullivan & Feinn, 2012).

2 The duration of the exercise thus varied in accord with the individual’s physiological capacity. Nonetheless, because the recruited participants all had similar physical fitness levels (i.e., recreationally active), the 5%-above-VT1 phase was rather brief and its duration fairly consistent among participants. No outliers were detected (see Statistical Analyses subsection).

3 The obtained optode array was the same for all participants because the *f*NIRS headset was rigid and did not facilitate customization of optode positioning.
