# Supplementary material for: Effects of auditory stimuli during exhaustive exercise on cerebral oxygenation and psychophysical responses
Source: Imaging Neurosci (Camb). 2026 Mar 20;4:IMAG.a.1166. doi: 10.1162/IMAG.a.1166 (PMC13007387; doi:10.1162/IMAG.a.1166)
Supplement: Supplementary Material 1 [file IMAG.a.1166_supp1.pdf]

Supplementary File 1: Selection of Music Tracks

Methods

Participants

Healthy volunteer adults were recruited among the corpus of University of ______ students. Eight non-musician participants (all French) engaged in the music selection procedure (four females, M_{age} = 30.0 years, SD = 3.1 years).

Selection of Music Tracks

Music tracks deemed to be motivational in an exercise context were selected using the following musical characteristics: tempo (120–124 beats per minute [BPM]), year of release (< 2 years), genre (pop songs that charted in France), time signature (four crotchet beats in a bar; i.e., common time), and language of the lyrics (English). Twenty tracks were selected on this basis (see Table 1). None of the selected tracks contained explicit lyrics or changes in tempo (i.e., there was an absence of accelerandos or rallentandos). The selected tracks were edited into 90-s excerpts using Audacity (2.3.3, http://audacity.sourceforge.net). Each excerpt started with the initiation of the main beat and contained at least one verse and one chorus.

Music-Rating Procedure

Task description

The music-rating exercise took place in a quiet room. Four participants were invited at a time and engaged in the music-rating panel (i.e., two sessions in total). Following a brief description of the music-rating exercise, participants read an information sheet and signed an informed consent form. Thereafter, they were asked to rate the 20 music excerpts. These were delivered using two speakers (Powered Studio Monitor HS8, YAMAHA®) placed in front of the participants (~3 m). For each excerpt, the research team ensured that participants completed three questionnaires (see below) before the next excerpt was presented. Participants were asked to remain silent throughout the music-rating session, which took ~1 h.

Assessment of musical qualities

For each of the 90-s excerpts, participants were administered the Brunel Music Rating Inventory-3 (BMRI-3; Karageorghis & Terry, 2011), the Affect Grid (Russell et al., 1989), and a familiarity scale attached to a 10-point Likert-type scale ranging from 1 (*not at all familiar*) to 10 (*extremely familiar*). The three questionnaires are presented in Annex 1. Note that the BMRI-3 was used to assess the motivational qualities of each track with reference to stationary cycling.

Data analyses

Data from the six subscales of the BMRI-3 were summed to compute a motivational quotient for each track. The mean valence and arousal scores were computed from the Affect Grid data. The mean familiarity score was computed.

Results

The ten tracks with the highest motivational quotient (min. = 24.88, max. = 28.25) were track nos. 1, 2, 3, 6, 8, 9, 10, 11, 19 and 20 (see Figure 1). The aforementioned tracks were all located in the top-right corner of the Affect Grid (pleasant–arousing; see Figure 2) and had low-to-moderate familiarity scores (min. = 2.50, max. = 6.38; see Figure 3), with exception of Track 1 and Track 6 which had very low familiarity scores (1.62 and 2.00, respectively). Consequently, these two tracks were removed from the list of tracks to be used in experimental trials and replaced by Track 5 and Track 13. This final list of tracks was of ~28 min duration (see Table 1).

References

Karageorghis, C. I., & Terry, P. C. (2011). *Inside sport psychology*. Human Kinetics.

Russell, J. A., Weiss, A., & Mendelsohn, G. A. (1989). Affect Grid: A single-item scale of pleasure and arousal. *Journal of Personality and Social Psychology, 57*(3), 493–502. https://citeseerx.ist.psu.edu/viewdoc/download?doi=10.1.1.1073.2381&rep=rep1&type=pdf

Table 1

Sample of Motivational Music Tracks

	Track Title	Artist(s)	Tempo (bpm)	Sex of Vocalist(s)	Length (min)	Home Key
1	Bad Decisions	Benny Blanco ft. BTS & Snoop Dogg	120	Male	02:53	C Major
**2**	**Young Right Now**	**Robin Schultz & Dennis Lloyd**	**123**	**Male**	**03:06**	**F Major**
**3**	**Take You Dancing (R3HAB Remix)**	**Jason Deruk**	**122**	**Male**	**02:56**	**D Major**
4	We Are The People	Martin Garrix ft. Bono & The Edge	120	Male	03:37	C Major
**5**	**Acapulco**	**Jason Derulo**	**122**	**Male**	**02:20**	**B♭ Major**
6	The Other Side (Oliver Heldens Remix)	SZA ft. Justin Timberlake	123	Female and Male	03:56	G Major
7	Célestial	Ed Sheeran	123	Male	03:29	D Major
**8**	**Belly Dancer**	**Imanbek**	**122**	**Male**	**02:31**	**C Major**
**9**	**Friday (Dopamine Re-Edit)**	**Riton ft. Mafasa & Hyperman**	**123**	**Female and Male**	**02:49**	**D Major**
**10**	**Never Going Home**	**Kungs**	**122**	**Male**	**02:50**	**C Major**
**11**	**Wasted Love**	**Ofenbach ft. Lagique**	**122**	**Male**	**02:20**	**F Minor**
12	Rain On Me	Lady Gaga ft. Ariana Grande	123	Female	03:02	A Major
**13**	**Head & Heart**	**Joel Corry ft. MNEK**	**123**	**Male**	**02:46**	**A Major**
14	Level Of Concern	Twenty One Pilots	122	Male	03:40	E Minor
15	The Business	Tiësto	120	Male	02:44	A Minor
16	Leave Before You Love Me	Marshmello	120	Male	02:35	G Major
17	Kiss Me	Dermot Kennedy	120	Male	03:49	C Minor
18	Lovefool	Twocolors	123	Male	03:10	F Major
**19**	**Take My Breath**	**The Weeknd**	**121**	**Male**	**03:40**	**A Major**
**20**	**Some Day (Felix Jaehn Remix)**	**Nea**	**120**	**Female**	**03:07**	**F Major**

Note. The music tracks selected for the experimental phase of the study are displayed in bold text.

Figure 1

Results From Administration of the Brunel Music Rating Inventory-3

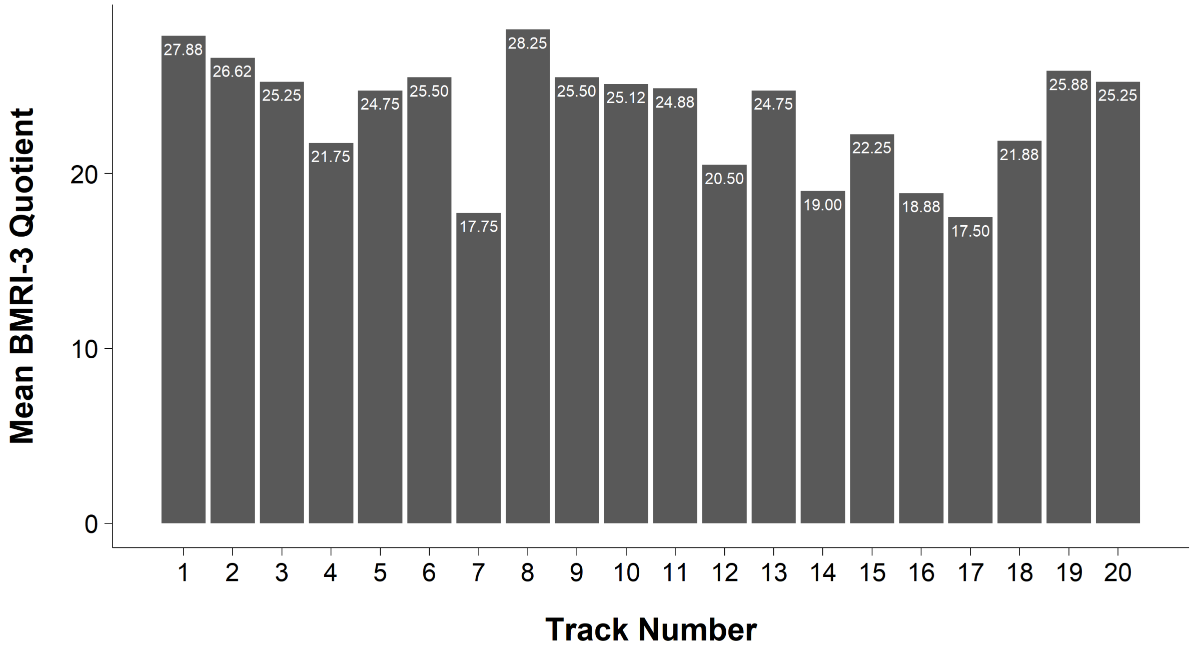

Note. BMRI-3 = Brunel Music Rating Inventory-3. The track numbers correspond with those presented in Table 1.

Figure 2

Results From Administration of the Affect Grid

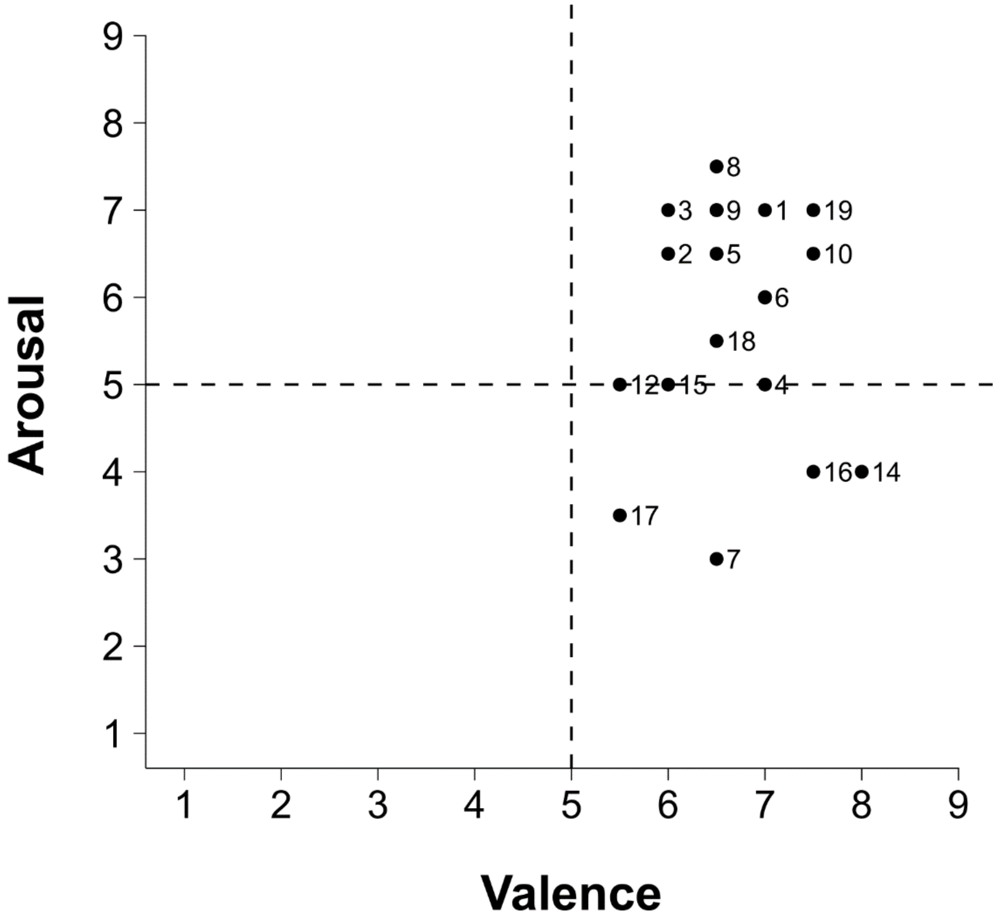

Note. Track nos 11, 13 and 20 are not displayed because Track 11 overlaps with Track 9, and Track 13 and Track 20 overlap with Track 6.

Figure 3

Results From Administration of the Familiarity Scale

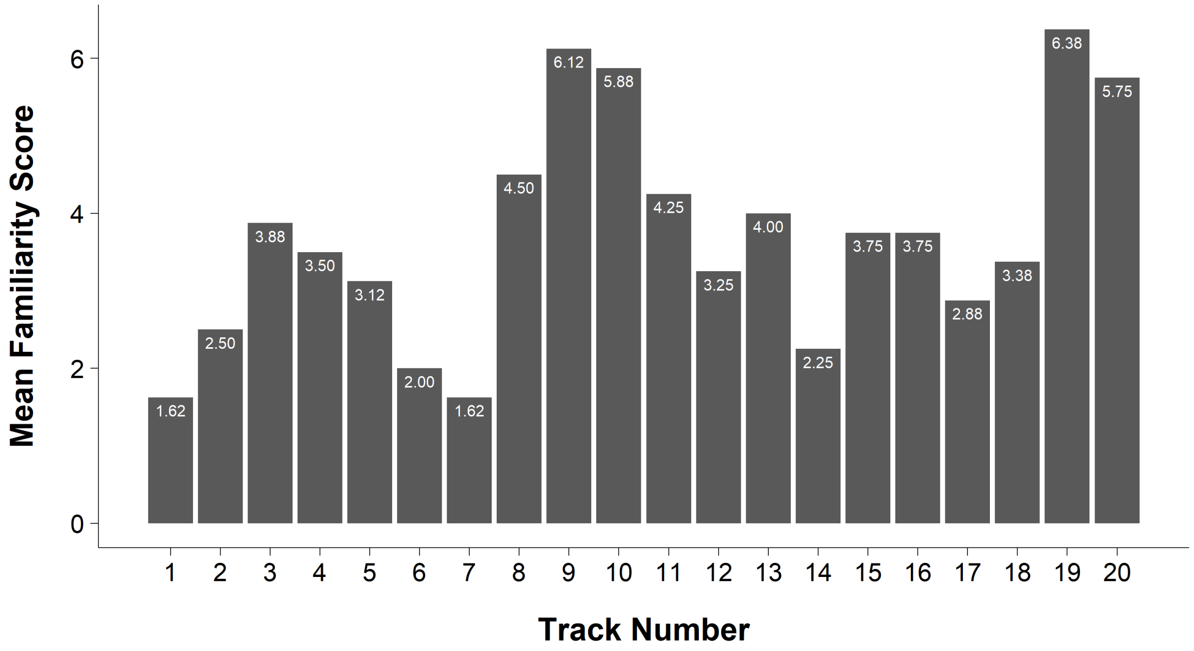

Annex 1

Brunel Music Rating Inventory-3

L'objectif de ce questionnaire est d'évaluer à quel point le morceau de musique que vous êtes sur le point d'entendre vous motiverait pendant une séance d'entraînement à vélo qui va durer environ 20 minutes. Ici, le mot « motiver » renvoie à une musique qui vous donnera envie de vous entraîner plus fort et plus longtemps. Pendant que vous écoutez le morceau de musique, indiquez à quel point vous êtes d'accord avec les affirmations listées ci-dessous en entourant l'un des numéros en dessous de chaque affirmation. Nous aimerions que vous fournissiez une réponse honnête pour chaque affirmation. Indiquez la réponse qui correspond le mieux à votre opinion et répondez le plus spontanément possible.

		**Fortement désaccord**						**Fortement d'accord**
1	Le rythme (i.e. la durée entre les notes) de cette musique me motiverait durant l'entraînement la séance.	1	2	3	4	5	6	7
2	Le style de cette musique (i.e. rock, dance, jazz, hip-hop, etc.) me motiverait durant la séance.	1	2	3	4	5	6	7
3	La mélodie (l'air) de cette musique me motiverait durant l'entraînement à vélo.	1	2	3	4	5	6	7
4	Le tempo (la vitesse) de cette musique me motiverait durant la séance.	1	2	3	4	5	6	7
5	Le son des instruments utilisés (i.e. guitare, synthétiseur, saxophone, etc.) me motiverait durant la séance.	1	2	3	4	5	6	7
6	Le beat (la qualité des basses) de cette musique me motiverait durant la séance.	1	2	3	4	5	6	7

Grille d'Affect

Les sentiments sont complexes. Ils se présentent sous toutes les formes et à tous les degrés. Les étiquettes que nous avons données ne sont que des points de repère pour vous aider à comprendre la grille des sentiments (i.e., stressant, énergique, enthousiaste, etc.). Lorsque vous utilisez la grille, placez un X n'importe où dans la grille pour indiquer la nuance exacte et l'intensité du sentiment que vous ressentez. Regardez l'ensemble de la grille pour vous faire une idée de la signification des différentes zones.

Veuillez s'il vous plait placer un X dans **UN SEUL** carré représentatif de la façon dont vous vous sentez présentement.

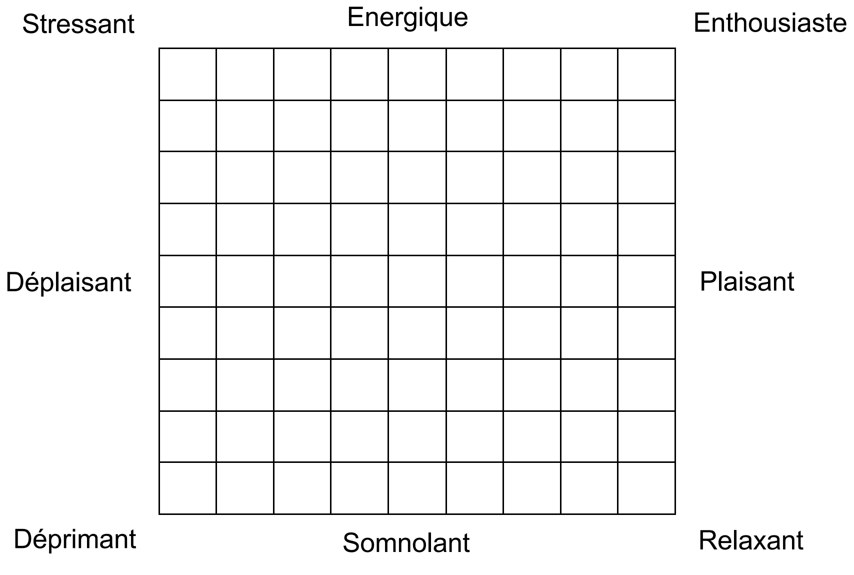

Echelle de Familiarité

Evaluez votre connaissance de cette chanson de 1 (*pas du tout familier*) à 10 (*extrêmement familier*).

Pas du tout									Extrêmement
1	2	3	4	5	6	7	8	9	10
