# Supplementary material for: Effects of auditory stimuli during exhaustive exercise on cerebral oxygenation and psychophysical responses
Source: Imaging Neurosci (Camb). 2026 Mar 20;4:IMAG.a.1166. doi: 10.1162/IMAG.a.1166 (PMC13007387; doi:10.1162/IMAG.a.1166)
Supplement: Supplementary Material 2 [file IMAG.a.1166_supp2.pdf]

Supplementary File 2: Determination of Ventilatory Threshold

During Session 1, participants will be administered an incremental cycle ergometer test to familiarise them with the experimental procedures and establish their first ventilatory threshold (VT1; i.e., the point during exercise at which breathing becomes laboured). This physiological index will be used to determine the individual exercise intensity (i.e., 5% above VT1) during the experimental phase.

Participants will be required to pedal at 63 rpm. They will start at 63 W (minimal basket weight = 1 kg) and the exercise intensity will be increased by 25 W (i.e., 0.4 kg) every 3 min (Karapetian et al., 2008) until volitional exhaustion (for a similar procedure, see e.g., Bigliassi et al., 2017; Barreto-Silva et al., 2018).

Figure 1

Graphic Representation of the Determination of the First Ventilatory Threshold

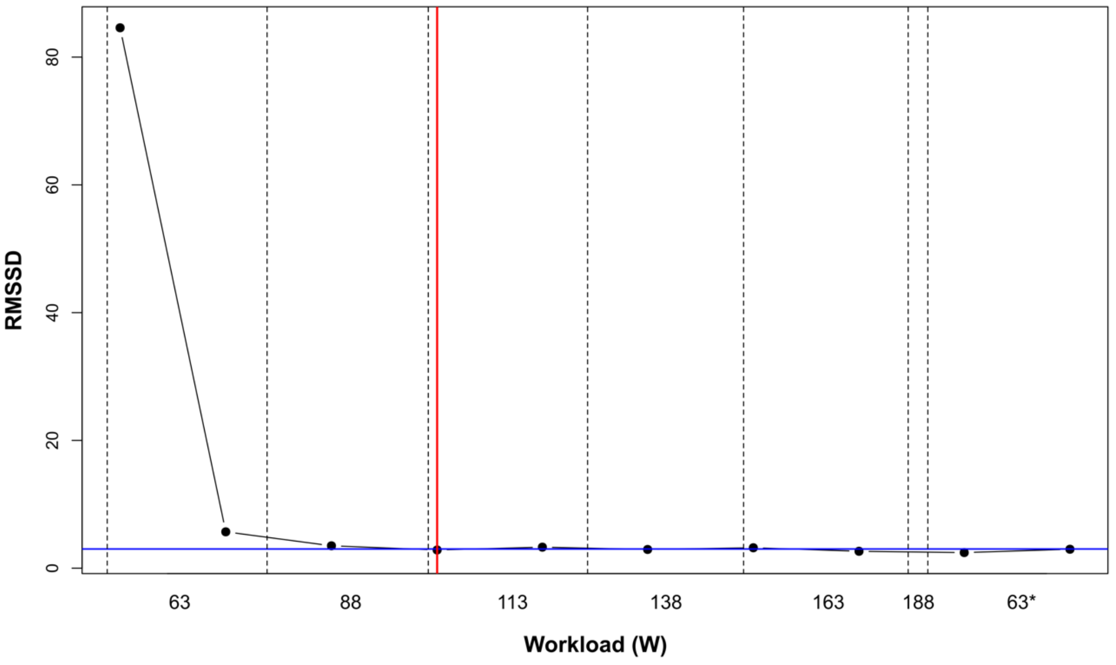

Note. The blue line indicates the 3 ms threshold. The red line indicates the first RMSSD value below the 3-ms threshold. * indicates the warm-down period. RMSSD = root mean square of successive differences between normal heartbeats; W = Watt.

A heart rate monitor (V800 Polar; H10 Polar strap) will be attached to the participant's chest to monitor the heart rate variability associated with increasing physiological load. R–R intervals will be monitored throughout the experiment using the HRV Logger app (window computation = 2 min; correction = workout). The root mean square of successive differences between normal heartbeats (RMSSD) will be computed. VT1 will be used to identify the precise exercise intensity at which the RMSSD value dropped below 3 ms (Queiroz et al., 2018; see Figure 1).

References

Barreto-Silva, V., Bigliassi, M., Chierotti, P., & Altimari, L. R. (2018). Psychophysiological effects of audiovisual stimuli during cycle exercise. *European Journal of Sport Science, 18*(4), 560–568. https://doi.org/10.1080/17461391.2018.1439534

Bigliassi, M., Karageorghis, C. I., Wright, M. J., Orgs, G., & Nowicky, A. V. (2017). Effects of auditory stimuli on electrical activity in the brain during cycle ergometry. *Physiology & Behavior, 177,* 135–147. https://doi.org/10.1016/j.physbeh.2017.04.023

Karapetian, G. K., Engels, H. J., & Gretebeck, R. J. (2008). Use of heart rate variability to estimate LT and VT. *International Journal of Sports Medicine, 29*(08), 652–657. https://doi.org/10.1055/s-2007-989423

Queiroz, M. G., Arsa, G., Rezende, D. A., Sousa, L. C. J. L., Oliveira, F. R., Araújo, G. G., & Cambri, L. T. (2018). Heart rate variability estimates ventilatory threshold regardless body mass index in young people. *Science & Sports, 33*(1), 39–46. https://doi.org/10.1016/j.scispo.2017.06.005
