# Supplementary material for: Effects of auditory stimuli during exhaustive exercise on cerebral oxygenation and psychophysical responses
Source: Imaging Neurosci (Camb). 2026 Mar 20;4:IMAG.a.1166. doi: 10.1162/IMAG.a.1166 (PMC13007387; doi:10.1162/IMAG.a.1166)
Supplement: Supplementary Material 3 [file IMAG.a.1166_supp3.pdf]

Supplementary File 3: Anatomical Specificity for Each *f*NIRS Channel

		MNI Coordinates (mm)			
Channel	Source–Detector	*x*	*y*	*z*	Specificity
1	Fpz–Fp1	-12	67	0	Frontopolar area (54.5%), orbitofrontal area (44.9%)
2	Fpz–Fp2	13	67	0	Frontopolar area (54.5%), orbitofrontal area (44.8%)
3	AF3–Fp1	-24	63	9	Frontopolar area (69.8%), orbitofrontal area (20.1%)
4	Fpz–AFz	1	64	14	Frontopolar area (87.5%)
5	AF4–Fp2	25	63	9	Frontopolar area (68.8%), orbitofrontal area (21.6%)
6	AF3–AFz	-12	62	23	Frontopolar area (75.8%), dlPFC (22.6%)
7	AF4–AFz	13	61	24	Frontopolar area (72.5%), dlPFC (24.9%)
8	AF3–F1	-23	52	32	dlPFC (80.5%), frontal polar area (16.9%)
9	Fz–AFz	2	50	39	dlPFC (61.7%), frontal polar area (20.3%), frontal eye fields (12.1%)
10	AF4–F2	22	52	33	dlPFC (77.9%), frontal polar area (18.4%)
11	F3–F1	-31	39	41	dlPFC (91.4%)
12	Fz–F1	-9	41	50	dlPFC (63.2%), frontal eye fields (34.7%)
13	Fz–F2	10	41	50	dlPFC (68.9%), frontal eye fields (28.9%)
14	F4–F2	30	40	41	dlPFC (90.8%)
15	CP3–C3	-52	-34	52	Primary somatosensory cortex (50.7%), supramarginal gyrus (43.3%),
16	CP4–C4	53	-35	52	Supramarginal gyrus (50.0%), primary somatosensory cortex (44.9%)
17	CP3–CP5	-57	-48	38	Supramarginal gyrus (65.5%), angular gyrus (14.4%)
18	CP3–CP1	-39	-48	60	Supramarginal gyrus (41.8%), somatosensory association cortex (27.0%), primary somatosensory cortex (22.0%)

Continued

Channel	Source–Detector	MNI Coordinates (mm)			Specificity
		x	*y*	*z*	
19	CP4–CP2	39	-49	60	Supramarginal gyrus (45.1%), somatosensory association cortex (25.3%)
20	CP4–CP6	58	-48	38	Supramarginal gyrus (69.0%), angular gyrus (12.8%)
21	CP3–P3	-46	-61	46	Angular gyrus (53.3%), supramarginal gyrus (29.8%), somatosensory association cortex (12.3%)
22	CP4–P4	46	-62	47	Angular gyrus (57.9%), supramarginal gyrus (25.0%), somatosensory association cortex (12.5%)
23	P5–P3	-46	-72	30	Angular gyrus (78.0%), visual area V3 (17.1%)
24	P6–P4	47	-72	30	Angular gyrus (80.0%), visual area V3 (15.1%)
25	Oz–O1	-14	-101	-2	Primary visual cortex (78.9%), secondary visual cortex (16.3%)
26	Oz–O2	-15	-99	-1	Primary visual cortex (67.6%), secondary visual cortex (26.7%)

Note. Information obtained from the fOLD toolbox (Morais et al., 2018). Only brain areas > 10% of specificity are reported. MNI = Montreal Neurological Institute; dlPFC = dorsolateral prefrontal cortex.

References

Morais, G. A. Z., Balardin, J. B., & Sato, J. R. (2018). *f*NIRS optodes' location decider (fOLD): A toolbox for probe arrangement guided by brain regions-of-interest. *Scientific Reports, 8,* Article 3341. https://doi.org/10.1038/s41598-018-21716-z
