# Supplementary material for: Effects of auditory stimuli during exhaustive exercise on cerebral oxygenation and psychophysical responses
Source: Imaging Neurosci (Camb). 2026 Mar 20;4:IMAG.a.1166. doi: 10.1162/IMAG.a.1166 (PMC13007387; doi:10.1162/IMAG.a.1166)
Supplement: Supplementary Material 4 [file IMAG.a.1166_supp4.pdf]

Supplementary File 4

Preregistration for *f*NIRS Research (PRE-*f*NIRS) Template (based on PRP-QUANT)

This preregistration template was developed by the authors of "Using preregistration as a tool for transparent *f*NIRS study design" (Philipp A. Schroeder, Christina Artemenko, Jessica E. Kosie, Helena Cockx, Katharina Stute, João Pereira, Franziska Klein, David M. A. Mehler), a guide that aims to aid researchers in writing a preregistration protocol. The preprint of this guide can be found here: https://osf.io/preprints/metaarxiv/zfawx. This template is based on the PRP-QUANT template and was adapted for study considerations specific to functional near-infrared spectroscopy (*f*NIRS) research.

This template offers guidance and examples for transparently generating a preregistration of an *f*NIRS study and analysis plan. The examples are based on a fictional *f*NIRS-EEG[1] finger-tapping study including the additional collection of electromyography (EMG), 9D inertial measurement unit (IMU), and short-distance channel (SDC) data. Whenever possible, the examples are inspired by the existing literature. Note that all examples mentioned here serve as guidance and do not necessarily describe a single study exhaustively. The tools, software, hardware and (preprocessing) algorithms mentioned in the examples are for illustrative purposes only and do not constitute any recommendations.

For researchers who intend to draft a Registered Report, we additionally recommend checking the guidelines at the respective target journal. For more information on Registered Reports, see: https://www.cos.io/rr

[1] The preprocessing pipeline for the EEG data is not covered in this template due to the focus on *f*NIRS preregistration. For preregistering an EEG study we recommend reading Paul et al. (2021) and Govaart et al.(2022).

Title

T1 Title
Effects of Auditory Stimuli During Submaximal Exercise on Cerebral Oxygenation

T2 Contributors, affiliations, and persistent IDs (recommend ORCID iD)
Blinded for peer review.

T3 Date of preregistration
24 January 2023

T4 Versioning information
https://doi.org/10.5281/zenodo.6261358

T5 Identifier
https://doi.org/10.5281/zenodo.6261358

T6 Estimated duration of project
9 months.

T7 IRB status (Institutional Review Board/Independent Ethics Committee/Ethical Review Board/Research Ethics Board)

The proposed protocol has been approved by the Comité de Protection de la Personne ethics committee of the University of [blinded for peer review] (ref. D2021-001).

T8 Conflict of interest statement

The authors have no competing financial interests to declare.

T9 Keywords

cerebral oximetry; cycling; physical activity; prefrontal activity; ventilatory threshold

T10 Data accessibility statement and planned repository

Pilot data are available on a public Zenodo repository (https://doi.org/10.5281/zenodo.6261358). All anonymised raw and processed data supporting the reported analyses will be archived in this repository at the point of Stage 2 submission. Participants will be informed on the participant information sheet that their anonymised data will be shared openly, as part-and-parcel of the publication process. This is made clear in our ethics-related documents (ref. D2021-001).

The raw *f*NIRS data will be shared in .sfnirs format (as recommended by the Society for functional Near Infrared Spectroscopy), the raw motion capture data in .tsv format, the raw physiological recording in .txt format, the head blood-volume pulse data in .csv format and the responses to questionnaires in .csv format. All the preprocessed data will be shared in .txt format. The full dataset will be converted to BIDS format containing the raw and proprocessed data for every measurement, and the metadata will be provided in the required .json and .tsv files. Anonymised demographic data will be shared as a .tsv file.

T11 Optional: Code availability

The code used to calculate the required sample size is available on a public Zenodo repository (https://doi.org/10.5281/zenodo.6261358). The study codes will be shared openly on the same public repository as part of the publication process.

T12 Optional: Standard lab practices
N/A

Abstract

(e.g., 200 words)

A1 Background

Asynchronous music has been commonly used to reduce perceived exertion and render the exercise experience more pleasant. Research has indicated that in-task asynchronous music can reallocate an individual's attentional focus to task-unrelated signals and increase the use of dissociative thoughts. Nonetheless, the brain mechanisms that underlie the purported benefits of music during exercise remain largely unknown due to the severe motion-related restrictions of popular neuroimaging techniques. *f*NIRS represents a non-invasive imaging method that is particularly suited to exercise-related protocols given its high tolerance to motion artifacts.

A2 Objectives and research questions

With use of *f*NIRS, the purpose of the proposed study will be to determine the point of onset of cerebral oxygenation decline during exercise and how this is influenced by the presence of asynchroneous (ambient) motivational music.

A3 Participants

Thirty-six adults in the age range 18–35 years, recreationally active, and apparently healthy will be recruited.

A4 Study method

A continuous-wave *f*NIRS system will be used to record the prefrontal, motor, and parietal haemodynamic responses of 24 participants who will perform a cycle-ergometry exercise protocol. The objective will be to test the hypothesis that brain oxygenation changes will be observed earlier when participants exercise with an audiobook or in silence, when compared with exposure to asynchronous music.
The results will shed light on the neurophysiological mechanisms that underlie the well-documented ergogenic and psychological effects of music.

Introduction

(no word limit)

I1 Theoretical background

This *f*NIRS technique will be used in the proposed study given its high tolerance for motion artefacts. In addition, the neurophysiological mechanisms that underlie the influence of attentional manipulation on tissue oxygenation during exercise can be investigated with an acceptable degree of temporal resolution (usually up to 10 Hz).

I2 Objectives and research question(s)

*f*NIRS is a technique that has proven to be effective in the examination of cortical oxygenation during exercise. The purpose of the proposed study will be to determine the point of onset of cerebral oxygenation decline during an incremental exercise protocol and how this is modulated by the presence of asynchronous music. More specifically, we will assess the effects of pleasurable auditory stimuli (i.e., music) on the cerebral oxygenation curve during a cycle ergometry exercise task.

I3 Hypothesis (H1, H2, …)

We hypothesise that the decrease in prefrontal (i.e., medial prefrontal cortex [mPFC] and dorsolateral prefrontal cortex [dlPFC]) HbO_2 concentration will be observed earlier under conditions in which participants exercise in silence or with an audiobook when compared with exposure to asynchronous motivational music (H_1). Exercise in silence or with an audiobook will lead to less prefrontal (H_2) and parietal (H_3) HbO_2 concentration when compared to exercising with music. These three hypotheses will be accepted if the statistical test is significant ($p < .020$) and the associated Cohen's $d > d_{SESOI}$. The null hypothesis is that there will be no significant condition-related change in HbO_2 concentration. For each research hypothesis, non-significant condition-related change in HbO_2 concentration will be followed-up with two one-sided tests (TOSTs) based on the smallest effect size of interest. If significant, the outcome will be interpreted as conclusive evidence for the absence of a meaningful effect.

As a sanity check for the effect of music exposure on prefrontal and parietal brain activity, we hypothesise that HbO_2 concentration in the occipital cortex will not differ among the experimental conditions (i.e., negative control; H_4). TOSTs will be used to test the absence of difference (i.e., equivalence testing), and the hypothesis will be confirmed if both t tests are significant.

I4 Exploratory research questions (if applicable; E1, E2, ...)

N/A

Method

M1 Time point of registration

Registration prior to creation of data.

M2 Proposal: Use of preexisting data (re-analysis or secondary data analysis)

N/A

Sampling Procedure and Data Collection

M3 Sample size, power and precision

Hypotheses and sampling plan are based on cerebral haemodynamic changes in the region of interest (ROI). To reduce multiple testing, we will restrict hypothesis testing to ROI analysis (i.e., across channels).

The sample size for the critical statistical test of each research hypothesis was calculated using R with the "pwr" and "TOSTER" packages (the code is available here: https://doi.org/10.5281/zenodo.6261358). The required sample size has been computed for paired-samples *t* tests, which are the critical statistical tests for the contrast of interest. The *f*NIRS results of Ozawa et al. (2019) were used as a parameter for H_1–H_2 across the mPFC. For H_1–H_2 across the dlPFC and H_3, the *f*NIRS results of Oh et al. (2018) were used. For H_4 the *f*NIRS results of Guérin et al. (2021) were used. For H_1–H_2, the power analysis indicated that 30 participants would be required for the mPFC ($d = 0.64$; $\alpha = .02$; $1\text{-}\beta = .90$) and nine participants for the dlPFC $d = 1.38$; $\alpha = .02$; $1\text{-}\beta = .90$. In addition, nine participants would be required for H_3 ($d = 1.37$; $\alpha = .02$; $1\text{-}\beta = .90$) and 36 participants for H_4 ($d = 0.62$; $\alpha = .02$; $1\text{-}\beta = .90$). Accordingly, a sample of 36 participants will be recruited for the proposed study.

M4 Participant recruitment, selection, and compensation

Participants will be recruited through local social networks (virtual and real) and advertisements on noticeboards. Volunteer adults will be eligible if in the age range 18–35 years, recreationally active, and apparently healthy. Recreationally active is defined as those who engage in 45–90 min of moderate-intensity exercise (3–6 metabolic equivalents [METs]) 2–4 times a week over the previous 6 months). To be included in the study, participants will need to have brought a recent

(under 12 months) medical certificate from their personal physician stating that they are fit to engage in high-intensity physical exercise. Participants will be excluded from the study if they self-report: (a) exercising > 5 times per week at moderate intensity, (b) incidents of motor dysfunction, (c) hearing deficiency, (d) epilepsy, or (e) head trauma (i.e., loss of consciousness for more than 5 min). They will be compensated for their time (i.e., €40 for the completion of all four trials).

M5 How will participant drop-out be handled?

If a participant does not complete all four trials, their full data set will be removed prior to the data analysis. In addition, a participant's entire data set will be removed prior to further analyses if all channels pertaining to at least one ROI are excluded following data-quality assessments. Any excluded participants will be replaced to ensure maintenance of $N = 36$.

M6 Masking of participants and researchers

All data collection will be performed by the same researcher. Instructions for participants will be drawn up and printed to ensure standardisation.

M7 Data cleaning and screening

To control for the quality of acquired *f*NIRS data, the power-spectral density of the raw *f*NIRS signals will be computed using Welch's estimation method, to check for the presence of the heart rate and respiration frequencies. The QT-NIRS toolbox will be used to identify channels with poor optical coupling though the computation of the scalp-coupling index (cardiac filter = 2.5–4 Hz; time window = 5 s; λ = 805 and and 830 nm). In addition, motion capture data will be used to detect shifts in the *f*NIRS headset within each experimental session.

M8 How will missing data be handled?

Excluded channels will be removed prior to data analysis. A participant's entire data set will be removed prior to further analyses if all channels pertaining to at least on ROI are excluded or if a shift in the *f*NIRS headset is detected.

M9 Instrumentation

The *f*NIRS data will be recorded by use of a continuous-wave *f*NIRS system (FOIRE-3000/16; Shimadzu, Kyoto). The system's light beam emanates from three lasers (class 1M) at three wavelengths of 780, 805 and 830 nm. The equipment contains 16 light sources (multicomponent glass bundle fibres) and 16 detectors (multi-alkali photomultipliers detectors). A 26-channel model

(11 sources and 15 detectors) will be designed in order to cover the brain ROIs over both the left and right hemispheres. Adjacent sources and detectors of infrared light are ~3 cm apart. The sampling frequency will be set at 10 Hz (i.e., temporal resolution of 100 ms).

Respiratory-rate monitoring will be facilitated by use of TSD201 respiratory effort transducer, connected to a MP150 Biopac device (Biopac Systems, Goleta, USA). This respiratory belt was placed around the chest wall, at the level of the sternum. The sampling frequency will be set to 250 Hz. Data acquisition was facilitated by the AcqKnowledge software that is included in the MP system. Heart rate will be assessed by means of a Polar system (H10 Polar strap) and the HRV Logger app (correction = workout).

To control for extra-cerebral noise, non-cortical haemodynamic responses will be collected by means of a photoplethysmograph sensor (Shimmer3 GSR+ unit; Shimmer, Dublin, Ireland) that will be attached to the participant's earlobe.

A motion capture technique (Qualisys MoCap, Götebord, Sweden) will be used to detect shifts in the *f*NIRS headset within each experimental session. Specifically, one passive marker will be taped to the participant's right temple and two markers to the *f*NIRS headset.

All data streams will be synchronised through markers send manually during the experiment.

M10 Optode array design and probe placement

In the proposed study, the brain regions of interest will be the bilateral dlPFC (Brodmann areas [BAs] 9 and 46), mPFC (BAs 10 and 11), lateral parietal cortex (BA 39 and 40) and primary visual cortex (BA 17). The fOLD toolbox will be used to guide the selection of optimal optode positioning with respect to the brain ROIs. Thus, a 26-channel model (11 sources and 15 detectors) was designed in order to cover the brain ROIs over both the left and right hemispheres. The obtained optode array will be the same for all participants because the *f*NIRS headset is rigid and does not facilitate customisation of optode positioning. The *f*NIRS headset holding the optodes will be placed on the participant’s head in accord with the International 10–20 system guidelines for standard electrode positions.

A system calibration will be conducted at the beginning of each experimental session by means of automatic adjustment using LabNIRS to verify that all optodes are emitting correctly. In case that the amount of light detected will be insufficient, the participant's hair will be pushed back beneath each problematic source–detector couple until data can be collected reliably.

Head details (i.e., head circumference, distance between the inion and nasion, distance between the left and right preauricular points) will be collected.

Conditions and design

M11 Type of study and study design

For each participant, the task will be executed under three conditions (taking place on different days, a minimum recovery period of 48 hours between session): asynchronous music, an audiobook control, and a no-audio control (i.e., within-subject design).

M12 Randomization of participants and/or experimental materials

Session 1 will entail screening, administration of questionnaires and protocol habituation. Sessions 2–4 will be administered in a fully counterbalanced order and comprise cycling (a) with asynchronous music (120–123 beats per minute [bpm]), (b) with an audiobook (audio control), (c) without any extraneous auditory stimuli (i.e., ambient noise control).

M13 Measured variables, manipulated variables, covariates

The mean haemodynamic response function (HRF) will be computed for each ROI (i.e., mPF, dlPFC, motor cortex, parietal cortex). For each trial, a polynomial regression will be fitted to the HRF. Thereafter, the decrease in cerebral oxygenation D will be defined as the time point at which the polynomial regression reaches its maximal value. To estimate the amplitude of changes in oxygenation during a trial, a linear regression will also be fitted to each HRF. The amount of cerebral oxygenation will be identified by the slope coefficient of the linear regression, referred to as β.

These two indices (i.e., D and β) will be computed on both HbO_2 and HHb. Because HbO_2 benefits from a better signal-to-noise ratio, only D_{HbO2} and β_{HbO2} will be used to support or refute the hypotheses. Nonetheless, HHb indices will also be analysed and the findings reported in the interests of transparency.

Prior to running analyses, we will examine the data and consider the inclusion of suitable covariates (e.g., BMI and a relative autonomy index from the BREQ-3) assuming that these meet the relevant covariate assumptions.

M14 Study materials

For the experimental conditions, the auditory stimulus (i.e., asynchronous music or audiobook) will be played to the participant through speakers from 1 min before the end of the warm-up session up to the point at which they reach volitional exhaustion.

During Session 1, participants will be administered several questionnaires relating to (a) socio-demographic and anthropometric details, (b) self-reported physical activity level (International Physical Activity Questionnaire; IPAQ), (c) motivation to engage in physical activity (Behavioural Regulations in Exercise Questionnaire; BREQ-3) and (d) tolerance of exercise intensity (Preference

for and Tolerance of the Intensity of Exercise Questionnaire, PRETIE-Q).

During Session 2–4, core affect (Feeling Scale [FS] and Felt Arousal Scale [FAS]), perceived exertion (Borg Category Ratio-10 scale, CR10) and attentional focus (Attention Scale) will be assessed during the cycle ergometer exercise. Physical activity enjoyment (Physical Activity Enjoyment Scale; PACES) and remembered pleasure (visual analogue scale) will be assessed at the end of each experimental session.

M15 Experimental design

The study will consist of four sessions. Session 1 will entail screening, administration of questionnaires, and protocol habituation. Specifically, the participant will read an information sheet, be afforded an opportunity to ask questions and sign an informed consent form. Participants will also perform an incremental VO_{2max} test on a cycle ergometer (Ergomedic 874E, Monark, Vansbro, Sweden) to determine a work rate representative of 5% above the first ventilatory threshold (VT1). Five percent above VT1 will be computed for each participant using the heart rate variability index of root mean square of successive differences. *f*NIRS data will not be collected during this first session.

Sessions 2–4 will be administered in a fully counterbalanced order and comprise cycling (a) with asynchronous music (120–123 beats per minute [bpm]), (b) with an audiobook (audio control), (c) without any extraneous auditory stimuli (i.e., ambient noise control). In each session, participants will undergo an exercise test on the cycle ergometer. Participants will cycle at a constant rate of 63 rpm (revolutions per minute) to avoid synchronisation of the pedal revolutions with the tempo of the music tracks (i.e., 120–123 bpm). After a 5-min warm up at 5% below VT1 and a 1-min transition phase performed at VT1, the resistance of the cycle ergometer will be increased so that the participant exercises at 5% above VT1. The session will be terminated when the participant is no longer able to maintain the prescribed pedal rate of 63 rpm for a period > 10 s. Thereafter, there will be a 3-min active warm down at 63 rpm at an intensity of 5% below VT1. The *f*NIRS technique will be used to monitor the brain activity of participants throughout each session.

M16 Other information (optional)

N/A

Analysis plan

(NOTE: If this varies by hypothesis, repeat analysis plan for each)

AP1 Criteria for post-data collection exclusion of participants (if any)

A participant's entire data set will be removed prior to further analyses if (a) the duration of the 5%-above-VT1 phase is unusually short or long (i.e., outlier, assessed with z scores > ± 3.29), (b) an *f*NIRS headset shift is detected, or (c) all channels pertaining to at least one ROI are excluded.

Only participants who have completed all four trials will be included for further analyses.

AP2 Criteria for post-data collection exclusions on trial level (if applicable)

NA

AP3 Criteria for post-data collection exclusions on channel level (if applicable)

A channel will be excluded prior to further analyses if (a) the heart rate frequency is not found in the frequency spectrum of *f*NIRS signals, (b) *f*NIRS signals are characterised by a scalp-coupling index < 0.8, or (c) motion artefacts will still be visible (i.e., high-frequency spikes and/or baseline shifts) in the *f*NIRS signals after motion artefacts correction.

AP4 Data preprocessing

Correction for motion artefacts will be performed using wavelet filtering (interquartile range = 0.5) in Homer 3 (v158.0; Massachusetts General Hospital, Boston, MA). The motion-corrected data will be visually inspected to ensure that the selected interquartile range value is well suited to the *f*NIRS data. In accord with the SPA-*f*NIRS guidelines, the recorded blood-pulse volume will be regressed from the collected *f*NIRS signals to account for non-cortical haemodynamic responses that are potential confounds. To reject both cardiac and breathing rates along with parts of Mayer oscillations, a lowpass filter set at 0.1 Hz will be applied.

AP5 Reliability analysis (if applicable)

N/A

AP6 Descriptive statistics

The mean and standard deviation for each dependent variable will be computed. For effect sizes that will be presented as Cohen's *d*, $d < 0.5$ will be considered as small, $d \geq 0.5$ as medium, and $d \geq 0.8$ as large. Where effect sizes will be presented as η^2_p, $\eta^2_p \geq .01$ will be considered as small, $\eta^2_p \geq .06$ as medium, and $\eta^2_p \geq .14$ as large.

AP7 Statistical models (provide for each hypothesis if varies)

The *f*NIRS dependent variables (i.e., D_{HbO2} and β_{HbO2}) will be averaged across ROIs and trials.

Data from the questionnaires will be analysed by means of one-way repeated-measures (multivariate) analysis of variance (RM [M]ANOVA; audio condition [music, audiobook, control]). The cardiorespiratory data will also be analysed and reported in a supplementary file. D_{HbO2} and β_{HbO2} will be analysed for each ROI by means of RM ANOVAs for H_1–H_3. The critical statistical tests used to confirm or disconfirm hypotheses will be the associated pairwise *t* tests from the post hoc analyses.

Normality will be checked in each cell of the analysis using the Shapiro–Wilk test. Where normality is violated, for nonself-reported data, a transformation will be used in accord with the nature of the distribution curve (e.g., log10, square root). Where Mauchly's test indicates violations of the sphericity assumption, Greenhouse–Geisser corrections will be applied to the *F* test. Bonferroni adjustments pairwise/multiple comparisons will be used where necessary to identify where differences lie.

Non-significant condition-related change in HbO_2 concentration will be followed-up with two one-sided tests (TOSTs) based on the smallest effect size of interest.

AP8 Inference criteria

In accord with the stipulations of the periodical *Cortex*, the significance level will be set at $p < .020$ for all analyses. Partial eta squared and Cohen's *d* effect sizes will be reported alongside each inferential analysis. The hypothesis will be accepted only if $d > d_{SESOI}$. Specifically, the small telescopes approach was used to determine the SESOI. The SESOI was set to the effect size that an earlier study would have had 33% power to detect. The *f*NIRS results of Oh et al. (2018) were used as parameters for H_1–H_4, with a one-tailed test for H_1–H_3, and a two-tailed test for H_4. The SESOI computations were performed using R (the code is available as supplementary material here: https://doi.org/10.5281/zenodo.6261358).

AP9 Exploratory analysis (optional)

N/A

AP10 Other information (optional)
N/A

Other information optional

O1 Other information (optional)
N/A

References

R1 References

Govaart, G., Schettino, A., Helbling, S., Mehler, D., Ngiam, W. X. Q., Moreau, D., ... & Paul, M. (2022). EEG ERP preregistration template. https://pure.mpg.de/rest/items/item_3430794/component/file_3430801/content

Guérin, S. M. R., Vincent, M. A., Karageorghis, C. I., & Delevoye-Turrell, Y. N. (2021). Effects of motor tempo on frontal brain activity: An *f*NIRS study. *NeuroImage, 230,* Article 117597. https://doi.org/10.1016/j.neuroimage.2020.117597473

Oh, S., Song, M., & Kim, J. (2018). Validating attentive locomotion training using interactive treadmill: An *f*NIRS study. *Journal of Neuroengineering and Rehabilitation, 15,* Article 122. https://doi.org/10.1186/s12984-018-0472-x549

Ozawa, S., Kanayama, N., & Hiraki, K. (2019). Emotion-related cerebral blood flow changes in the ventral medial prefrontal cortex: An NIRS study. *Brain and Cognition, 134,* 21–28. https://doi.org/10.1016/j.bandc.2019.05.001552

Paul, M., Govaart, G. H., & Schettino, A. (2021). Making ERP research more transparent: Guidelines for preregistration. *International Journal of Psychophysiology, 164,* 52–63. https://doi.org/10.1016/j.ijpsycho.2021.02.016

This **Preregistration for fNIRS Research (PRE-fNIRS)** template (version 1) was created by Philipp A. Schroeder, Christina Artemenko, Jessica E. Kosie, Helena Cockx, Katharina Stute, João Pereira, Franziska Klein, and David M. A. Mehler alongside the paper "Using preregistration as a tool for transparent fNIRS study design" (preprint available at: https://osf.io/preprints/metaarxiv/zfawx). This document is an adapted and extended version of the Psychological Research Preregistration-Quantitative (aka PRP-QUANT) Template, version 2 (available at https://www.psycharchives.org/). The original template was developed by a task force composed of members of the American Psychological Association (APA), the British Psychological Society (BPS), the German Psychological Society (DGPs), the Center for Open Science (COS), and the Leibniz Institute for Psychology (ZPID).

This work is licensed under the CC BY 4.0 license. Thus, you are free to share and adapt the content, given that you attribute the source and indicate if changes were made. To cite the fNIRS preregistration template, use this reference:
Schroeder, P.A., Artemenko, C., Kosie, J.E., Cockx, H., Stute, K., Pereira, J., Klein, F., & Mehler, D.M.A. (2022). Using preregistration as a tool for transparent fNIRS study design. https://osf.io/preprints/metaarxiv/zfawx.
The associated project page on the Open Science Framework can be found via https://osf.io/hb4um/

To receive a timestamp and a DOI (digital object identifier), submit your preregistration protocol, for example, to **PsychArchives** via https://pasa.psycharchives.org/ or to OSF via https://osf.io/, preferably as PDF.
