# Supplementary material for: Effects of auditory stimuli during exhaustive exercise on cerebral oxygenation and psychophysical responses
Source: Imaging Neurosci (Camb). 2026 Mar 20;4:IMAG.a.1166. doi: 10.1162/IMAG.a.1166 (PMC13007387; doi:10.1162/IMAG.a.1166)
Supplement: Supplementary Material 5 [file IMAG.a.1166_supp5.pdf]

Supplementary File 5: Cardiorespiratory Results

Data Screening and Diagnostics

Data screening indicated no univariate outliers. The normality assumption was not met for both heart rate ($p < .001$) and respiration rate ($p = .011$). An ordered quantile normalization transformation (Peterson & Cavanaugh, 2020) was applied to remedy this.

Heart and Respiratory Rates

The oneway RM ANOVA on heart rate showed no significant main effect of condition, $F(1.52, 44.09) = 1.60$, $p = .216$, $\eta_p^2 = .05$. The oneway RM ANOVA on respiratory rate showed a significant main effect of condition, $F(2, 70) = 6.47$, $p = .003$, $\eta_p^2 = .16$; nonetheless, the post hoc tests did not reach significance ($M_{music} = 0.71$, $SD_{music} = 0.16$; $M_{audiobook} = 0.69$, $SD_{audiobook} = 0.11$; $M_{control} = 0.65$, $SD_{control} = 0.12$). Overall, the results indicated that heart and respiratory rates were not influenced by condition (see Figure 1).

Figure 1

Heart Rate and Respiratory Rate

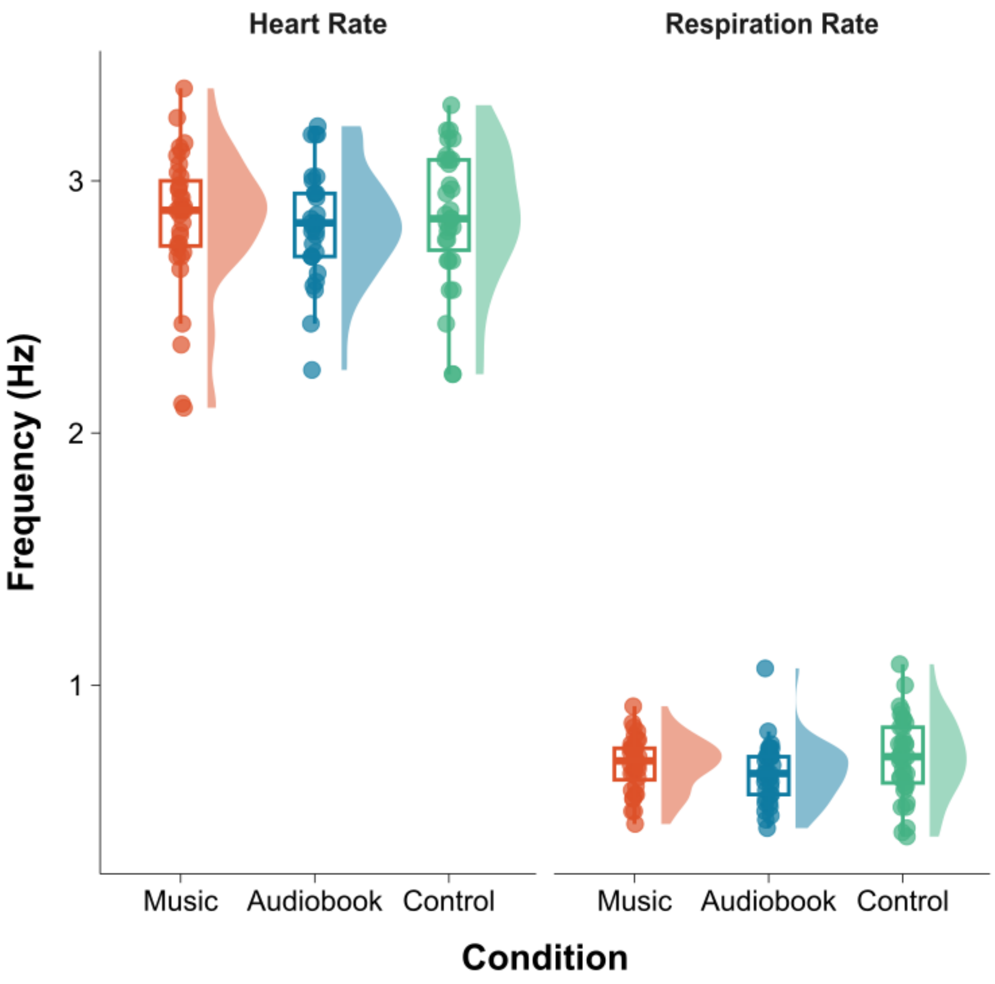

Note. Box plots and probability density functions or mean points and 95% confidence intervals are displayed for each condition. Each dot represents an individual participant.

References

Peterson, R. A., & Cavanaugh, J. E. (2020). Ordered quantile normalization: A semiparametric transformation built for the cross-validation era. *Journal of Applied Statistics*, *47*(13–15) 2312–2327. https://doi.org/10.1080/02664763.2019.1630372
