# Supplementary material for: Effects of auditory stimuli during exhaustive exercise on cerebral oxygenation and psychophysical responses
Source: Imaging Neurosci (Camb). 2026 Mar 20;4:IMAG.a.1166. doi: 10.1162/IMAG.a.1166 (PMC13007387; doi:10.1162/IMAG.a.1166)
Supplement: Supplementary Material 6 [file IMAG.a.1166_supp6.pdf]

Supplementary File 6: Photoplethysmography Results

In order to regress the blood-pulse volume recorded with the photoplethysmograph sensor, an independent component (IC) analysis was conducted on the raw *f*NIRS signal for each index (i.e., HbO_2 and HHb). Pearson's correlation coefficient was computed between each of the first 20 ICs and the photoplethysmography signal. The IC exhibiting the highest correlation coefficient was removed from the *f*NIRS signal (i.e., the signal was reconstructed excluding that IC). Details of the correlation coefficients between the removed IC and each designated *f*NIRS index can be found in Table 1.

Table 1

Descriptive Statistics for Photoplethysmography Results

Index	Minimum	Maximum	Median	*M*	*SD*	*SE*
HbO_2	.025	.502	.148	.174	.101	.010
HHb	.029	.492	.107	.124	.080	.008

Note. HbO_2 = oxygenated hemoglobin; HHb = deoxygenated hemoglobin.
