# Supplementary material for: Effects of auditory stimuli during exhaustive exercise on cerebral oxygenation and psychophysical responses
Source: Imaging Neurosci (Camb). 2026 Mar 20;4:IMAG.a.1166. doi: 10.1162/IMAG.a.1166 (PMC13007387; doi:10.1162/IMAG.a.1166)
Supplement: Supplementary Material 7 [file IMAG.a.1166_supp7.pdf]

Supplementary File 7: Deoxyhemoglobin Results

In contrast to the approach used for oxygenated hemoglobin (HbO_2; see the main manuscript), the decrease in cerebral oxygenation D_i for deoxygenated hemoglobin (HHb) was defined as the time point at which the polynomial regression reached its *minimal* value.

Data Screening and Diagnostics

Data screening indicated that there were four univariate outliers. These were associated with $\beta_{HHb,mPFC}$ ($k = 1$), $\beta_{HHb,dlPFC}$ ($k = 1$), and $\beta_{HHb,lPC}$ ($k = 2$) measures. The outliers were adjusted using a winsorization procedure (see Sullivan et al., 2021) until they came within the range $\pm$ -3.29 < z > $\pm$ 3.29 (see Tabachnick & Fidell, 2018).

The normality assumption was not met for D_{HbO2} ($p < .001$) in each of the two brain regions of interest, and proved resistant to various transformations (e.g., rank-based inverse normal transformation, square-root, reflect and log/square root, and Yeo–Johnson transformation). Accordingly, the nonparametric Friedman rank sum test was employed. Finally, normality tests also indicated that β_{HbO2}—for each of the three brain regions of interest—exhibited instances of non-normality ($p < .001$), and ordered quantile normalization transformations (Peterson & Cavanaugh, 2020) were applied to remedy this.

Decrease in Cerebral Oxygenation

The Friedman test performed on D_{HbO2} in the mPFC showed no significant main effect of condition, $\chi^2(2) = 0.34$, $p = .844$, $W < .01$. The Friedman test performed on D_{HbO2} in the dlPFC was also nonsignificant, $\chi2(2) = 7.45$, $p = .024$, $W = .10$. Overall, it was evident that the delay in cerebral oxygenation decrease before volitional exhaustion was not affected by condition.

Amplitude of Activation

The oneway RM ANOVA on β_{HbO2} in the mPFC showed no significant main effect of condition, $F(2, 20) = 0.25$, $p = .779$, $\eta_p^2 = .02$, as did the oneway RM ANOVA on β_{HbO2} in the dlPFC, $F(1.27, 11.43) = 0.03$, $p = .906$, $\eta_p^2 < .01$, and the oneway RM ANOVA on β_{HbO2} in the lateral parietal cortex, $F(2, 70) = 0.23$, $p = .797$, $\eta_p^2 < .01$. Overall, the results indicated that activation of the mPFC, dlPFC, and lateral parietal cortex was not influenced by condition.

References

Peterson, R. A., & Cavanaugh, J. E. (2020). Ordered quantile normalization: A semiparametric transformation built for the cross-validation era. *Journal of Applied Statistics*, *47*(13–15) 2312–2327. https://doi.org/10.1080/02664763.2019.1630372

Sullivan, J. H., Warkentin, M., & Wallace, L. (2021). So many ways for assessing outliers: What really works and does it matter? *Journal of Business Research*, *132*, 530–543. https://doi.org/10.1016/j.jbusres.2021.03.066

Tabachnick, B. G., & Fidell, L. S. (2018). *Using multivariate statistics* (7th ed.). Pearson.
