# Supplementary material for: Effects of auditory stimuli during exhaustive exercise on cerebral oxygenation and psychophysical responses
Source: Imaging Neurosci (Camb). 2026 Mar 20;4:IMAG.a.1166. doi: 10.1162/IMAG.a.1166 (PMC13007387; doi:10.1162/IMAG.a.1166)
Supplement: Supplementary Material 8 [file IMAG.a.1166_supp8.pdf]

Supplementary File 8: Additional Questionnaires Analyses

Self-Reported Physical Activity Level

The participants displayed heterogeneous levels of physical activity (see Figure S1). They engaged most in walking (i.e., a low-intensity activity; *M* = 3,757.6 metabolic equivalent [MET], *SD* = 3,057.5), followed by vigorous-intensity activities (*M* = 3,185.6 MET, *SD* = 4,255.3), and moderate-intensity activities (*M* = 2,407.1, *SD* = 3,886.4).

Motivation to Engage in Physical Activity

The Behavioural Regulations in Exercise Questionnaire (BREQ-3; Markland & Tobin, 2004) was used to assess participants' motives for exercise. The exercise motives that were most endorsed by the participants were Identified (*M* = 2.94, *SD* = 0.77), Integrated (*M* = 2.78, *SD* = 1.03), and Intrinsic forms of regulation (*M* = 3.35, *SD* = 0.63). There was a high degree of heterogeneity evident with respect to Introjected Regulation (*M* = 1.92, *SD* = 0.93). The exercise motives least endorsed by the participants were External Regulation (*M* = 0.33, *SD* = 1.25) and Amotivation (*M* = 0.05, *SD* = 0.13). Overall, it was evident that the sample of participants was characterized by exercise motives representing a relatively high degree of self-determination.

Figure S1

Self-Reported Physical Activity Level

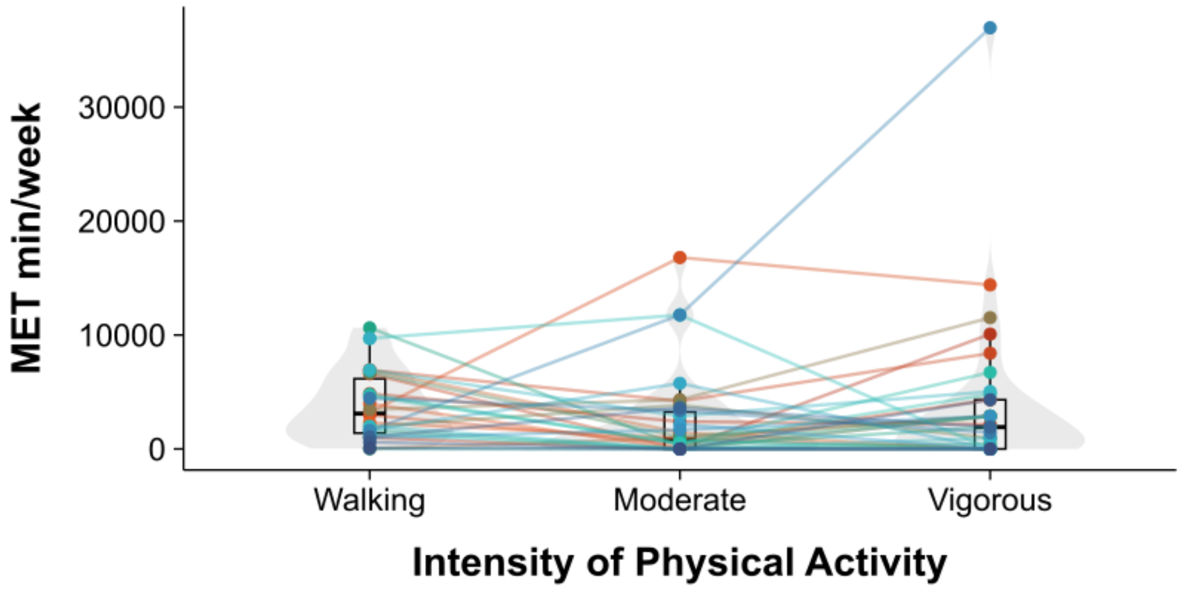

Note. Box plots and probability density functions are displayed for each intensity of physical activity. Each dot represents an individual participant. MET = metabolic equivalent.

Figure S2

Motivation to Engage in Physical Activity

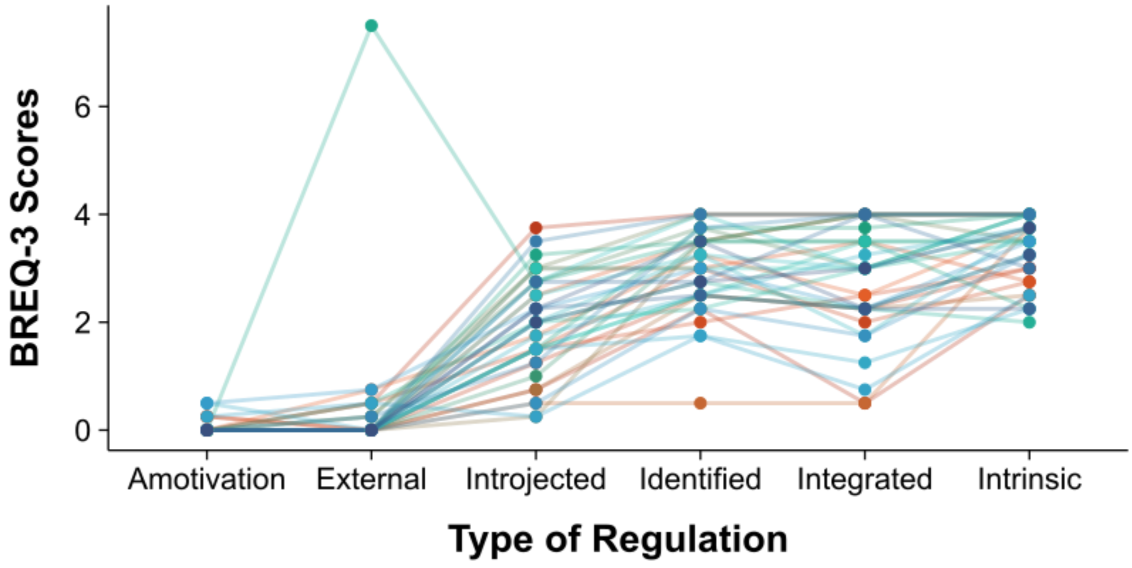

Note. Each dot represents an individual participant. BREQ-3 = Behavioural Regulations in Exercise Questionnaire.

Tolerance of Exercise Intensity

The participants exhibited heterogeneous levels of tolerance of exercise intensity (*M* = 3.17; *SD* = 0.88) and preference for exercise intensity (*M* = 3.17; *SD* = 0.80; see Figure S3).

Figure S3

Tolerance of and Preference for Exercise Intensity

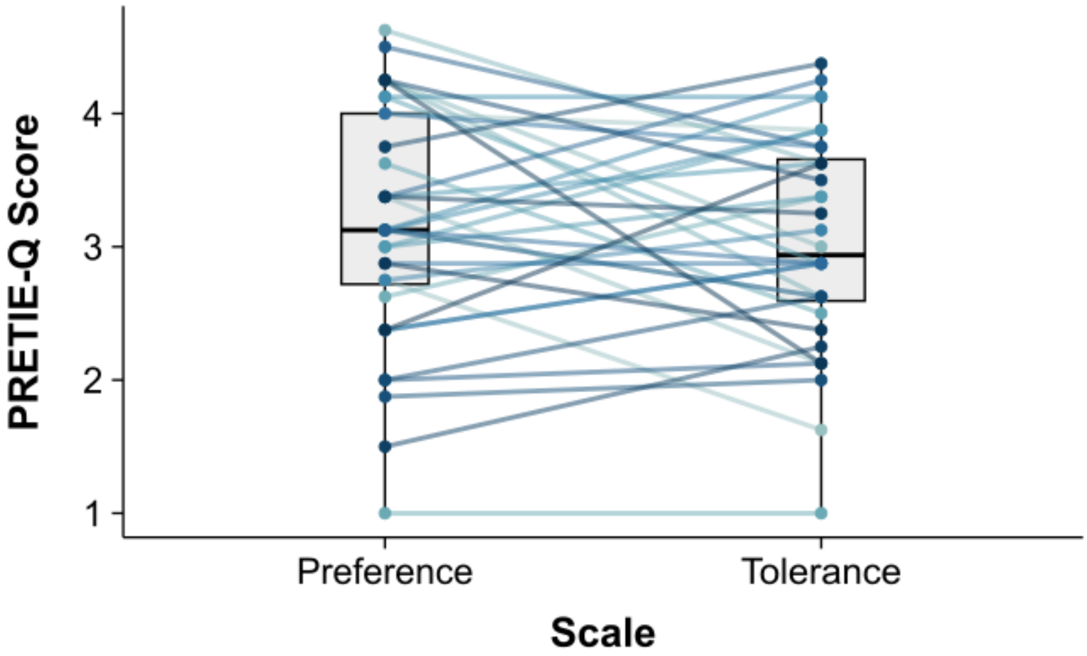

Note. Box plots are displayed for each scale. Each dot represents an individual participant. PRETIE-Q = Preference for and Tolerance of the Intensity of Exercise Questionnaire.

References

Markland, D., & Tobin, V. (2004). A modification to the Behavioural Regulation in Exercise Questionnaire to include an assessment of amotivation. *Journal of Sport & Exercise Psychology*, *26*(2), 191–196.
