# Supplementary material for: Effects of auditory stimuli during exhaustive exercise on cerebral oxygenation and psychophysical responses
Source: Imaging Neurosci (Camb). 2026 Mar 20;4:IMAG.a.1166. doi: 10.1162/IMAG.a.1166 (PMC13007387; doi:10.1162/IMAG.a.1166)
Supplement: Supplementary Material 9 [file IMAG.a.1166_supp9.pdf]

**Supplementary File 9: Additional Figures**

**Figure S1**

*Decrease in Cerebral Oxygenation in the Medial Prefrontal Cortex*

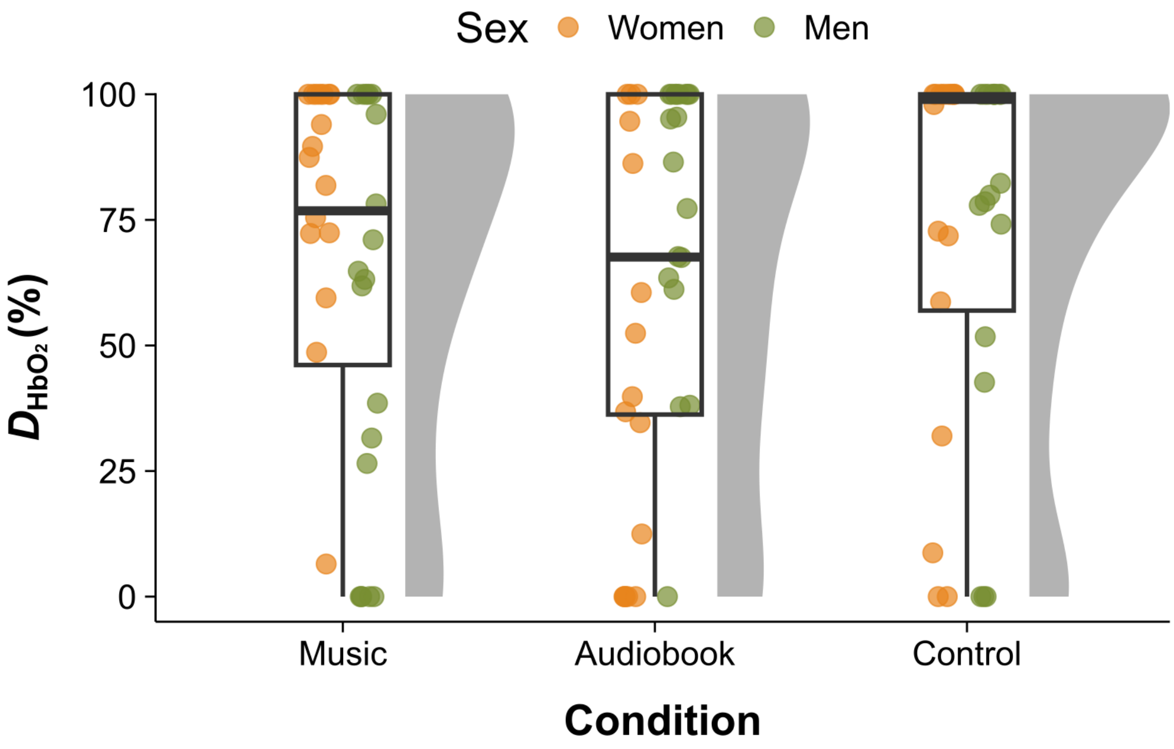


*Note.* Box plots and probability density functions are displayed for each condition and sex. Each dot represents an individual participant. $HbO_2$ = oxygenated hemoglobin.

**Figure S2**

*Decrease in Cerebral Oxygenation in the Dorsolateral Prefrontal Cortex*

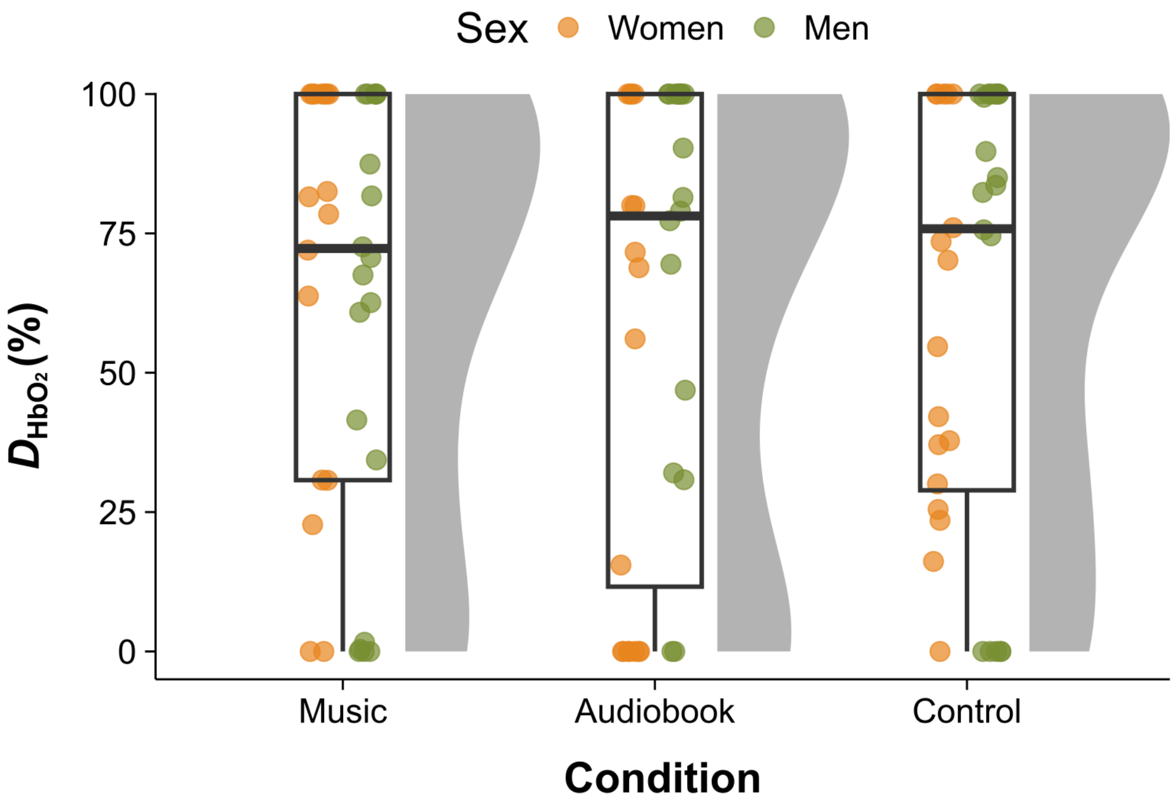


*Note.* Box plots and probability density functions are displayed for each condition and sex. Each dot represents an individual participant. $HbO_2$ = oxygenated hemoglobin.

**Figure S3**

*Amplitude of Activation in the Medial Prefrontal Cortex*

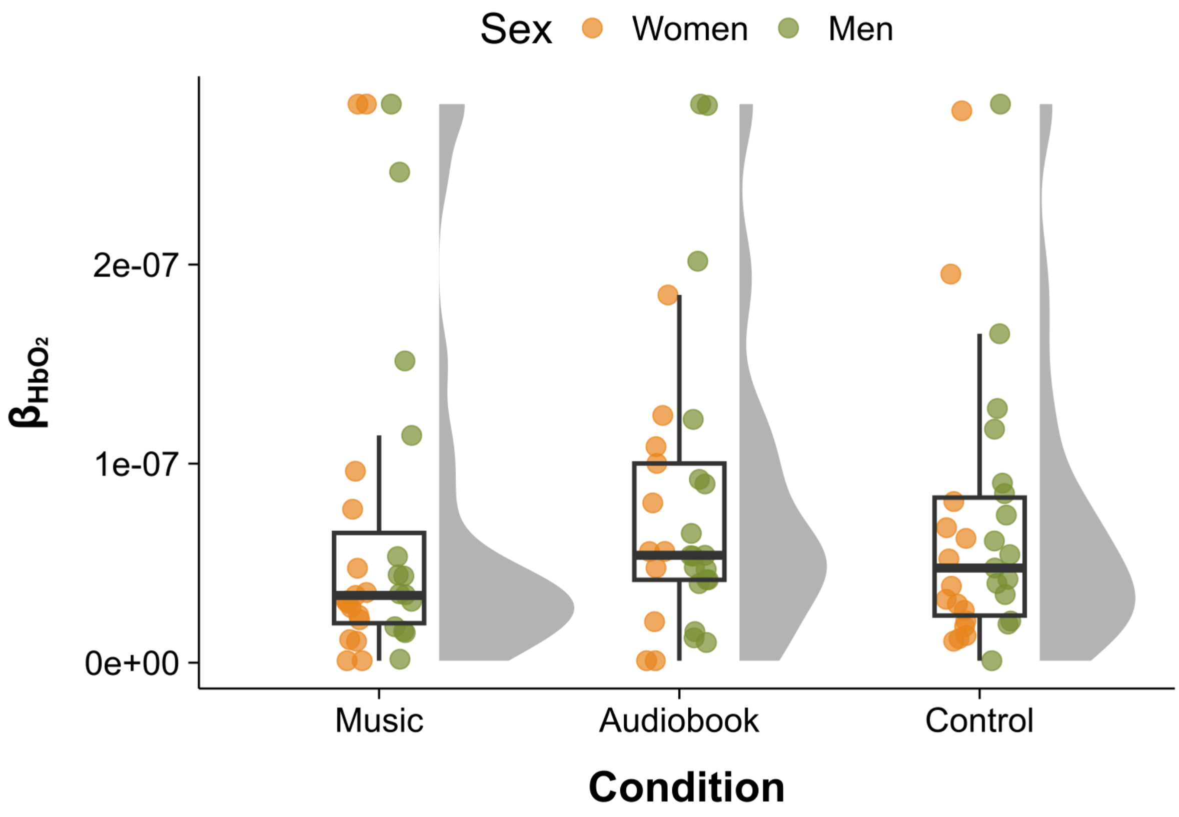


*Note.* Non-normalized data with an outlier removed. Box plots and probability density functions are displayed for each condition and sex. Each dot represents an individual participant. $HbO_2$ = oxygenated hemoglobin.

**Figure S4**

*Amplitude of Activation in the Dorsolateral Prefrontal Cortex*

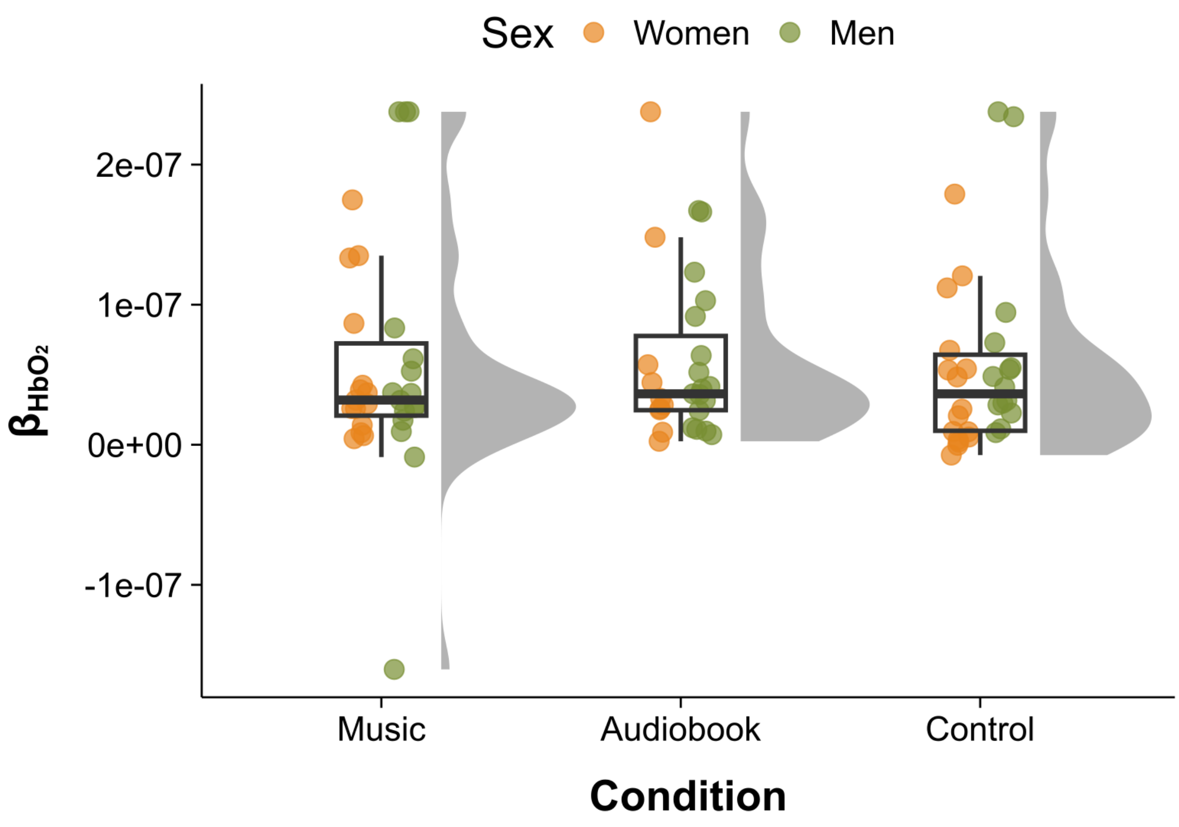


*Note.* Non-normalized data with an outlier removed. Box plots and probability density functions are displayed for each condition and sex. Each dot represents an individual participant. $HbO_2$ = oxygenated hemoglobin.

**Figure S5**

*Amplitude of Activation in the Lateral Parietal Cortex*

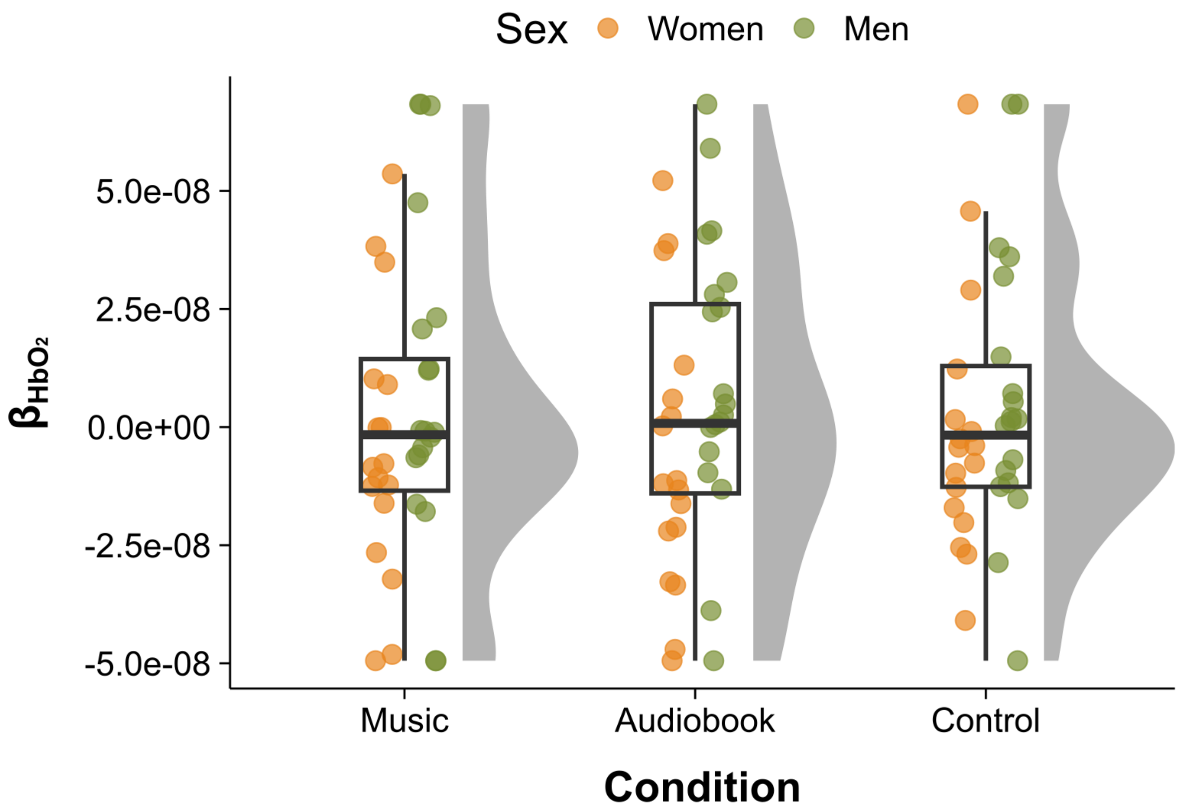


*Note.* Non-normalized data with outliers removed. Box plots and probability density functions are displayed for each condition and sex. Each dot represents an individual participant. $HbO_2$ = oxygenated hemoglobin.

**Figure S6**

*Amplitude of Activation in the Occipital Cortex*

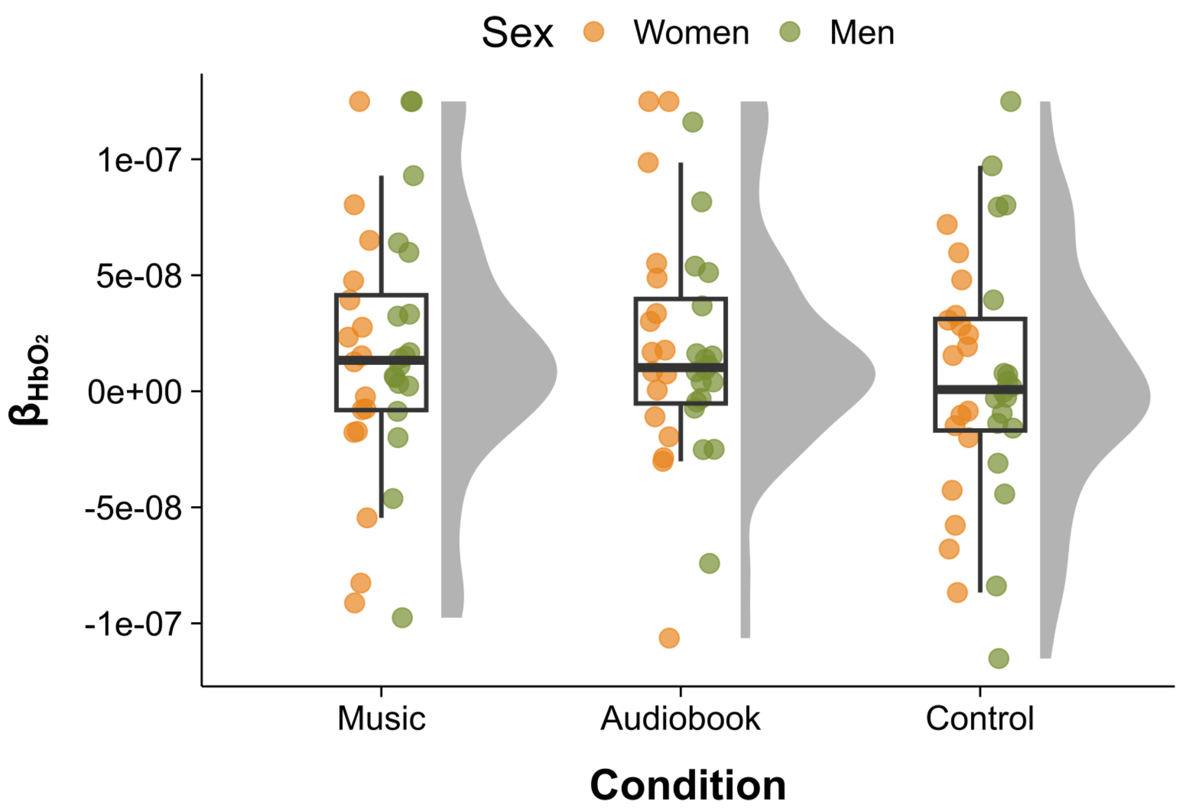


*Note.* Non-normalized data with an outlier removed. Box plots and probability density functions are displayed for each condition and sex. Each dot represents an individual participant. $HbO_2$ = oxygenated hemoglobin.
