# Supplementary material for: Effects of auditory stimuli during exhaustive exercise on cerebral oxygenation and psychophysical responses
Source: Imaging Neurosci (Camb). 2026 Mar 20;4:IMAG.a.1166. doi: 10.1162/IMAG.a.1166 (PMC13007387; doi:10.1162/IMAG.a.1166)
Supplement: Supplementary Material 10 [file IMAG.a.1166_supp10.pdf]

Supplementary File 10: Variability in the Hemodynamic Responses

Figure S1

Individual Findings Pertaining to Three Representative Participants

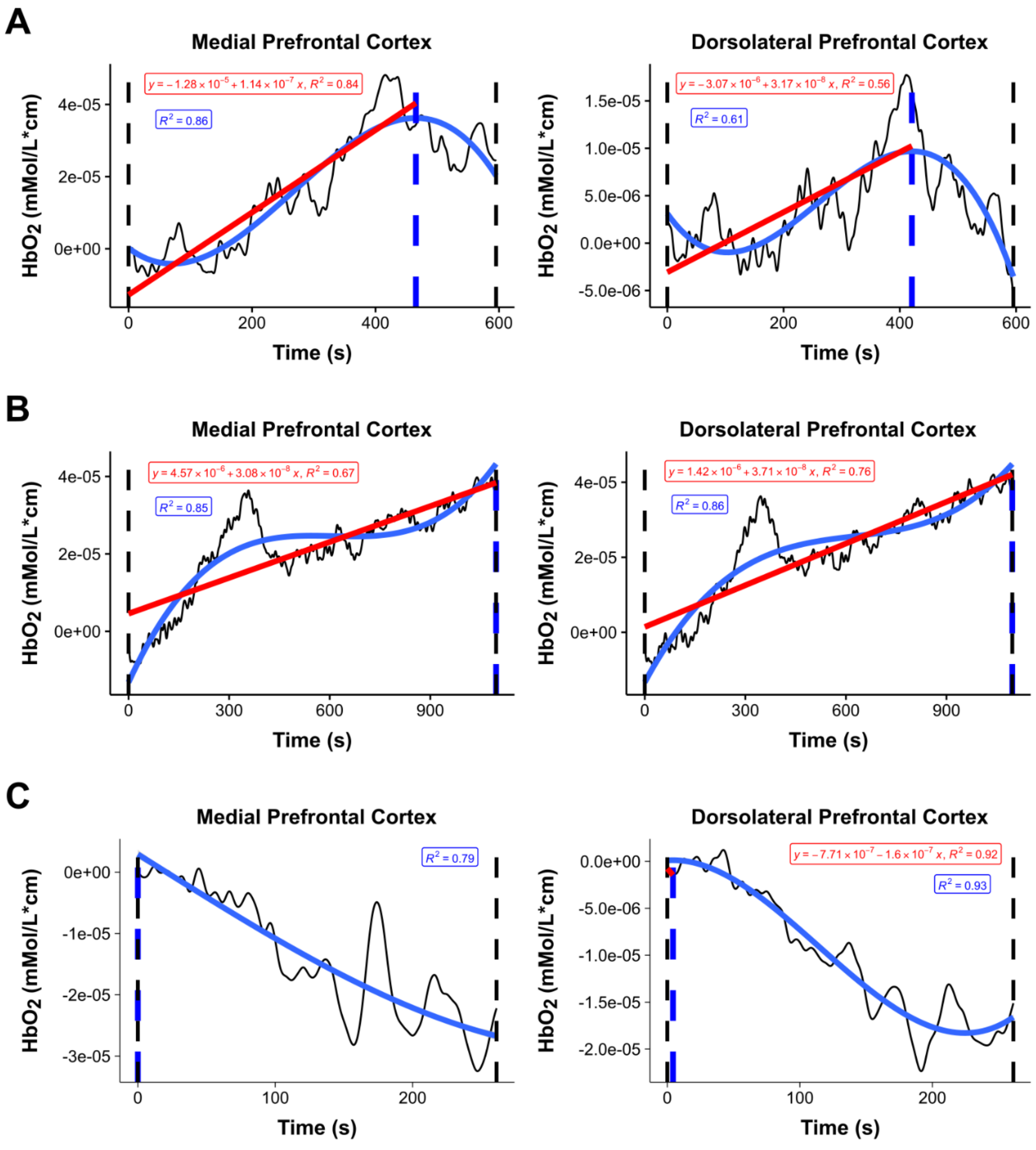

Note. Panel A: Mean hemodynamic response function in the prefrontal areas for the music condition for a participant displaying the expected pattern of activation. Panel B: Mean hemodynamic response function in the prefrontal areas for the music condition for a participant displaying a pattern of linear *increase* in activation. Panel C: Mean hemodynamic

response function in the prefrontal areas for the music condition for a participant displaying a pattern of linear *decrease* in activation. Dotted black lines indicate the beginning and end of the 5%-above-volitional-exhaustion phase. The polynomial regression is displayed in blue. The dotted blue line indicates the time point at which the maximal value of the polynomial regression was reached. The linear regression is displayed in red. Note that 0 on the *x* axis corresponds with the beginning of the 5%-above-volitional-exhaustion phase. HbO_2 = oxygenated hemoglobin.
