# Supplementary material for: Effects of auditory stimuli during exhaustive exercise on cerebral oxygenation and psychophysical responses
Source: Imaging Neurosci (Camb). 2026 Mar 20;4:IMAG.a.1166. doi: 10.1162/IMAG.a.1166 (PMC13007387; doi:10.1162/IMAG.a.1166)
Supplement: Supplementary Material 11 [file IMAG.a.1166_supp11.pdf]

Supplementary File 11: Time Series of Cardiorespiratory Measures

Figure S1

Heart Rate Data

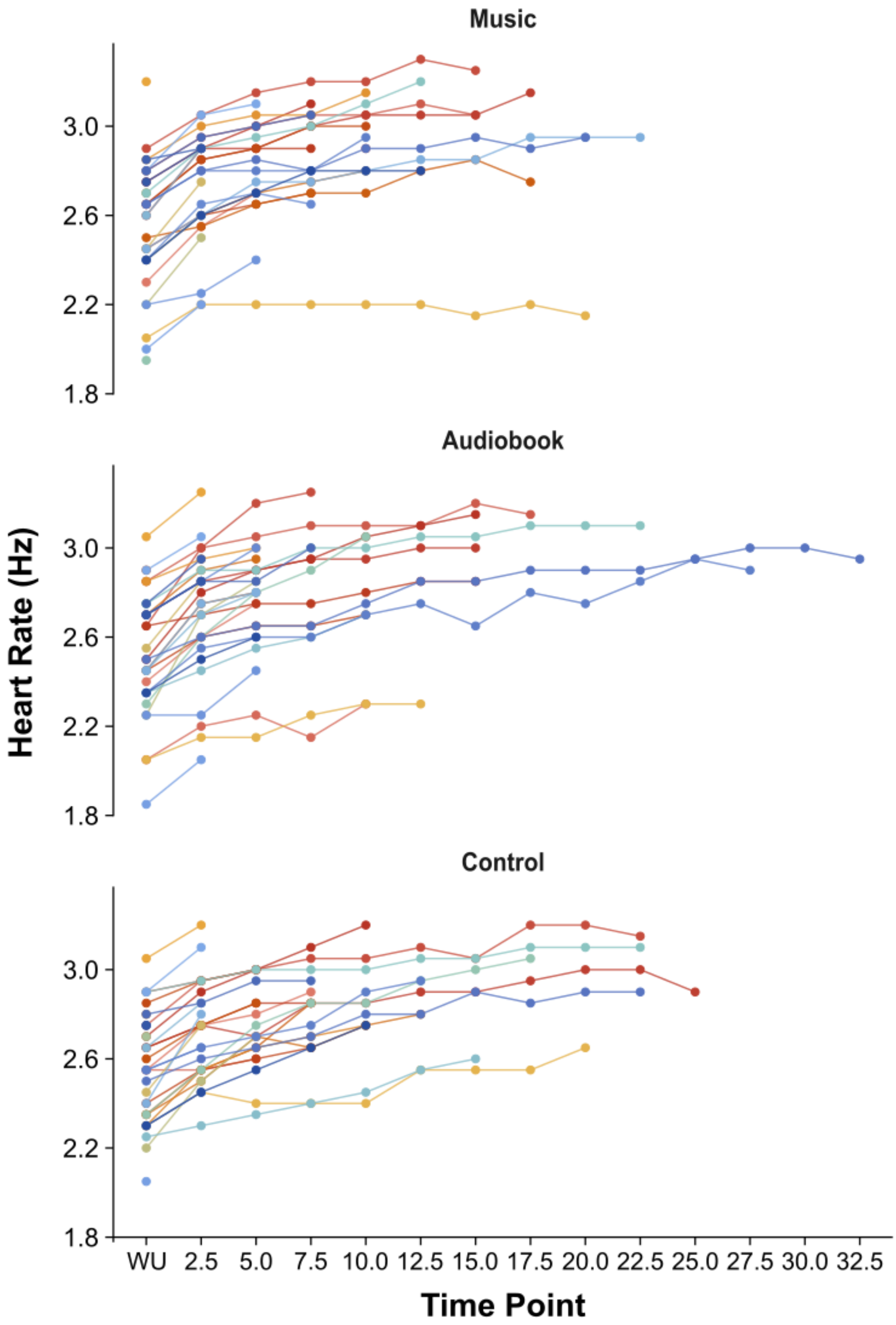

Note. Heart rate during warm up and for each 2.5-min interval, in each condition. Each color represents an individual participant. WU = warm up.

Figure S2

Respiratory Rate Data

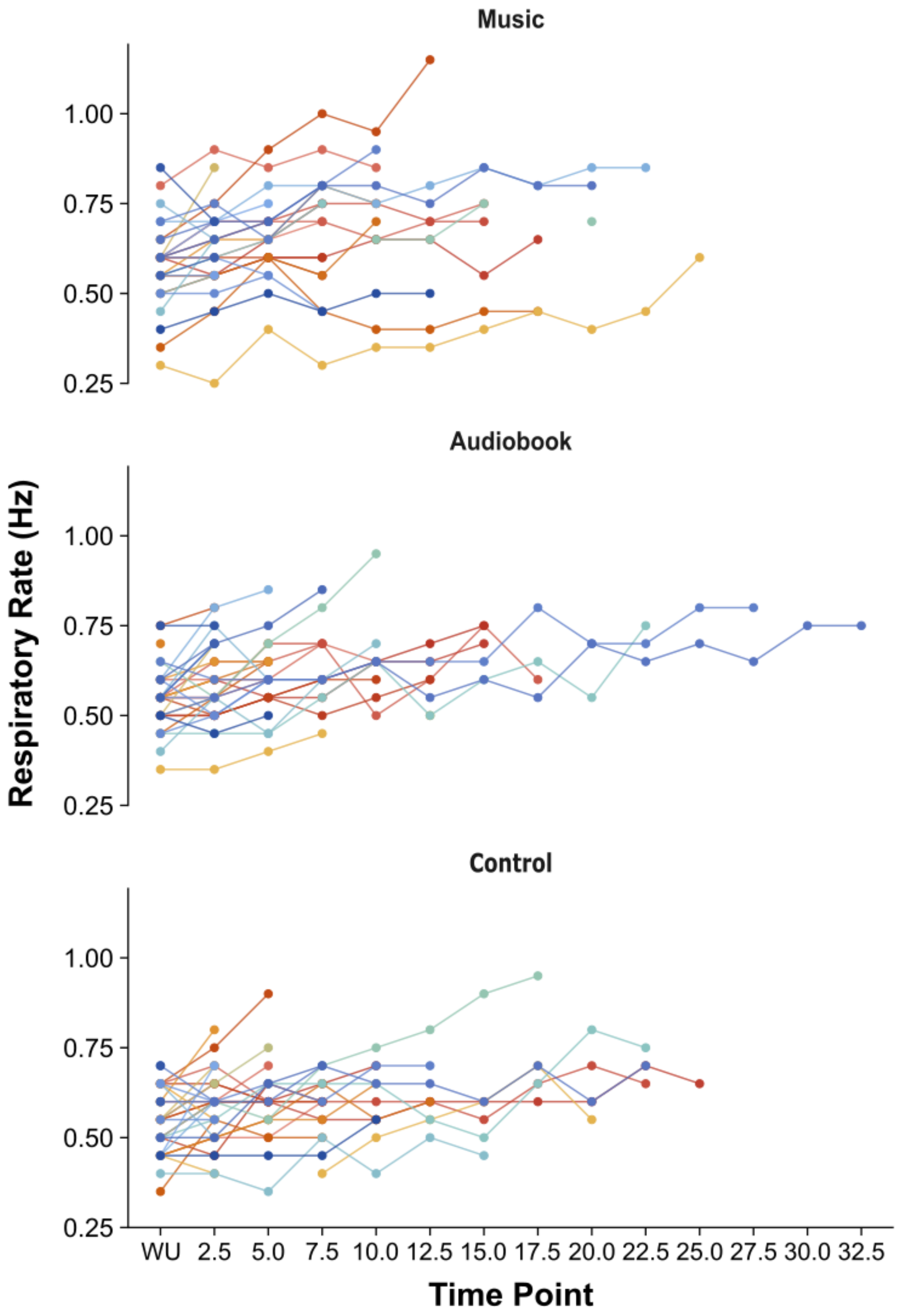

Note. Respiratory rate during warm up and for each 2.5-min interval, in each condition. Each color represents an individual participant. WU = warm up.
