# Supplementary material for: Effects of auditory stimuli during exhaustive exercise on cerebral oxygenation and psychophysical responses
Source: Imaging Neurosci (Camb). 2026 Mar 20;4:IMAG.a.1166. doi: 10.1162/IMAG.a.1166 (PMC13007387; doi:10.1162/IMAG.a.1166)
Supplement: Supplementary Material 12 [file IMAG.a.1166_supp12.pdf]

Supplementary File 12: Exercise Intensity

Figure S1

In-Task Measures of Perceived Exertion

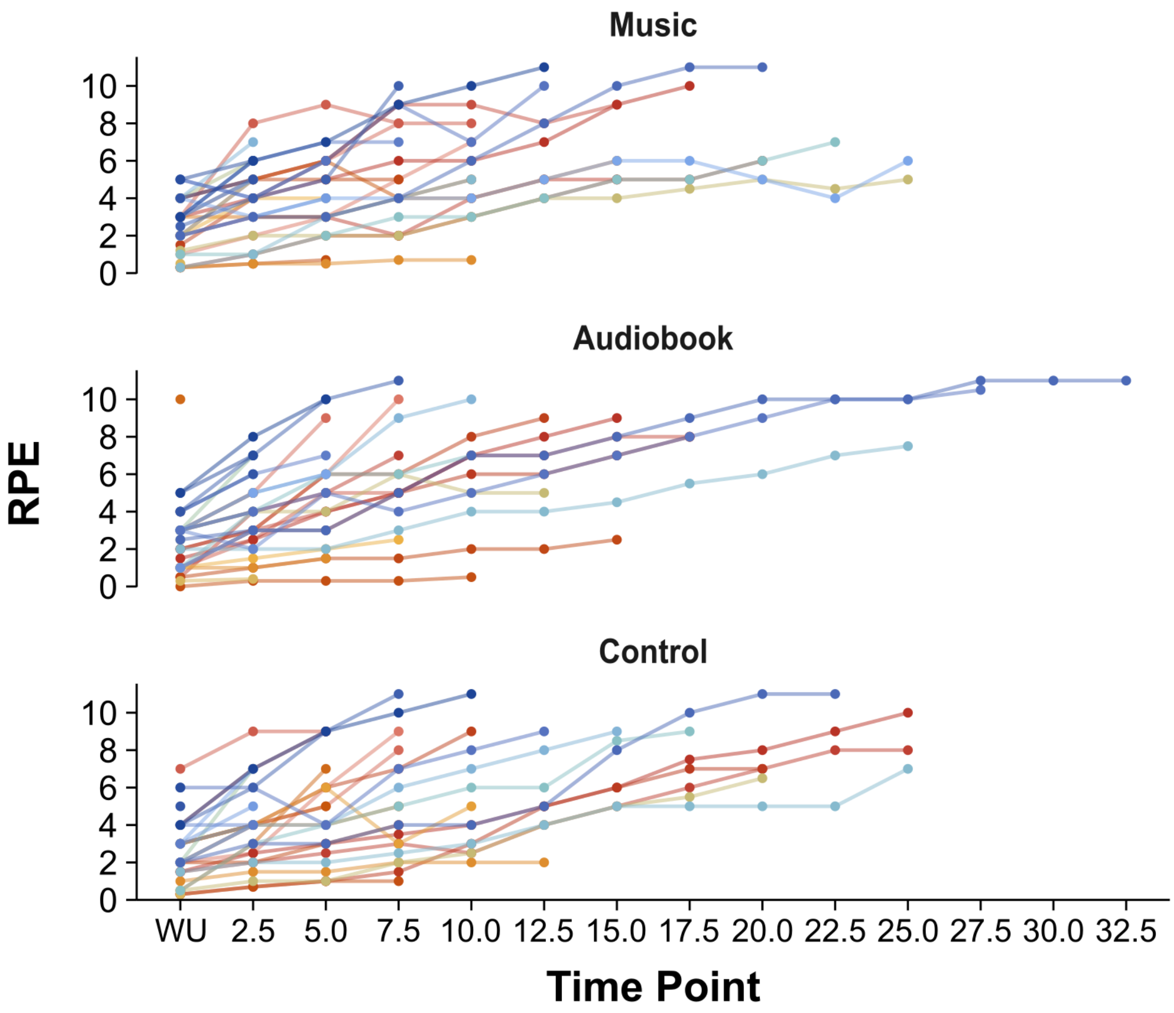

Note. Rating of perceived exertion during warm up and for each 2.5-min interval, in each condition. Each color represents an individual participant. RPE = rating of perceived exertion; WU = warm up.

Figure S2

Individual Workload During the 5%-Above-Ventilatory-Threshold Phase

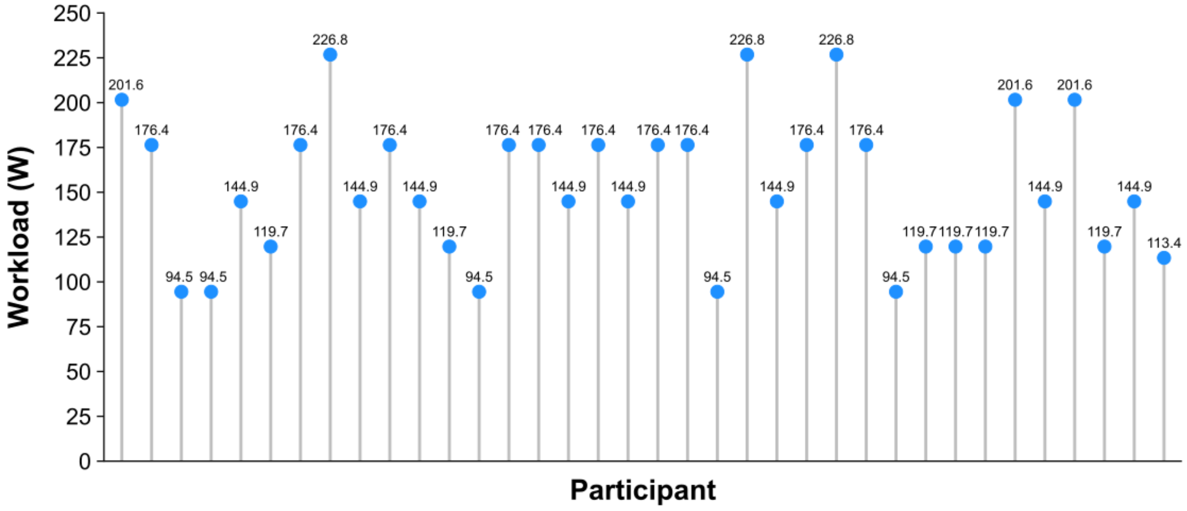

Note. Each dot represents an individual participant.
